# Analysis of gene expression from systemic lupus erythematosus synovium reveals myeloid cell-driven pathogenesis of lupus arthritis

**DOI:** 10.1038/s41598-020-74391-4

**Published:** 2020-10-15

**Authors:** Erika L. Hubbard, Michelle D. Catalina, Sarah Heuer, Prathyusha Bachali, Robert Robl, Nicholas S. Geraci, Amrie C. Grammer, Peter E. Lipsky

**Affiliations:** 1 RILITE Research Institute and AMPEL BioSolutions LLC, 250 West Main Street Suite #300, Charlottesville, VA 22902 USA; 2 Present Address: EMD Serono Research and Development Institute, 45 A Middlesex Turnpike, Billerica, MA 01821 USA; 3 Present Address: The Jackson Laboratory, Tufts Graduate School of Biomedical Sciences, 600 Main Street, Bar Harbor, ME 04609 USA; 4 Present Address: Profiler Business Unit, Genedata Inc, 1 Cranberry Hill, Lexington, MA 02421 USA

**Keywords:** Computational biology and bioinformatics, Immunology, Rheumatology

## Abstract

Arthritis is a common manifestation of systemic lupus erythematosus (SLE) yet understanding of the underlying pathogenic mechanisms remains incomplete. We, therefore, interrogated gene expression profiles of SLE synovium to gain insight into the nature of lupus arthritis (LA), using osteoarthritis (OA) and rheumatoid arthritis (RA) as comparators. Knee synovia from SLE, OA, and RA patients were analyzed for differentially expressed genes (DEGs) and also by Weighted Gene Co-expression Network Analysis (WGCNA) to identify modules of highly co-expressed genes. Genes upregulated and/or co-expressed in LA revealed numerous immune/inflammatory cells dominated by a myeloid phenotype, in which pathogenic macrophages, myeloid-lineage cells, and their secreted products perpetuate inflammation, whereas OA was characterized by fibroblasts and RA of lymphocytes. Genes governing trafficking of immune cells into the synovium by chemokines were identified, but not in situ generation of germinal centers (GCs). Gene Set Variation Analysis (GSVA) confirmed activation of specific immune cell types in LA. Numerous therapies were predicted to target LA, including TNF, NFκB, MAPK, and CDK inhibitors. Detailed gene expression analysis identified a unique pattern of cellular components and physiologic pathways operative in LA, as well as drugs potentially able to target this common manifestation of SLE.

## Introduction

Systemic lupus erythematosus (SLE) is a complex autoimmune disease in which loss of self-tolerance gives rise to pathogenic autoantibodies causing widespread inflammation and tissue damage^1^. Lupus arthritis (LA) is a common manifestation of SLE with 65–95% of lupus patients reporting joint involvement during the course of their disease^2^.

Despite the high frequency of LA, an understanding of the underlying pathogenic mechanisms remains incomplete. Cytokines, such as IL-6, and anti-dsDNA autoantibodies are thought to play a role^3–5^. Other autoantibodies including anti-ribonucleoprotein, anti-histone, and anti-proliferating cell nuclear antigen have been implicated in LA along with evidence of increased C-reactive protein (CRP) and erythrocyte sedimentation rate^4,6,7^.

The lack of a better understanding of the nature of LA relates to the difficulty of obtaining tissue samples and the absence of relevant and reliable animal models. Despite this, in most recent clinical trials of potential lupus therapies, arthritis is a principal manifestation and the success of a tested therapy can depend on its ability to suppress synovial inflammation. Therefore, it is essential to understand more about the pathogenic mechanisms operative in LA.

One way to evaluate the pathologic processes involved in LA is to analyze gene expression profiles in the affected synovium. Previous work analyzed global gene expression profiles and histology of SLE, rheumatoid arthritis (RA), and osteoarthritis (OA) synovium^8,9^ to begin to elucidate the inflammatory mechanisms in each disease and focused on the type 1 interferon pathway in LA. Here, we expand upon these studies by applying contemporary bioinformatic techniques to assess the only gene expression data set available to gain additional insight into the pathogenesis of LA. Using a multipronged, bioinformatic and systems biology approach, we provide an expanded view of SLE synovitis that might serve as the basis to identify new targeted therapies.

## Results

### Bioinformatic and pathway analysis of LA and OA gene expression

**Figure 1:**
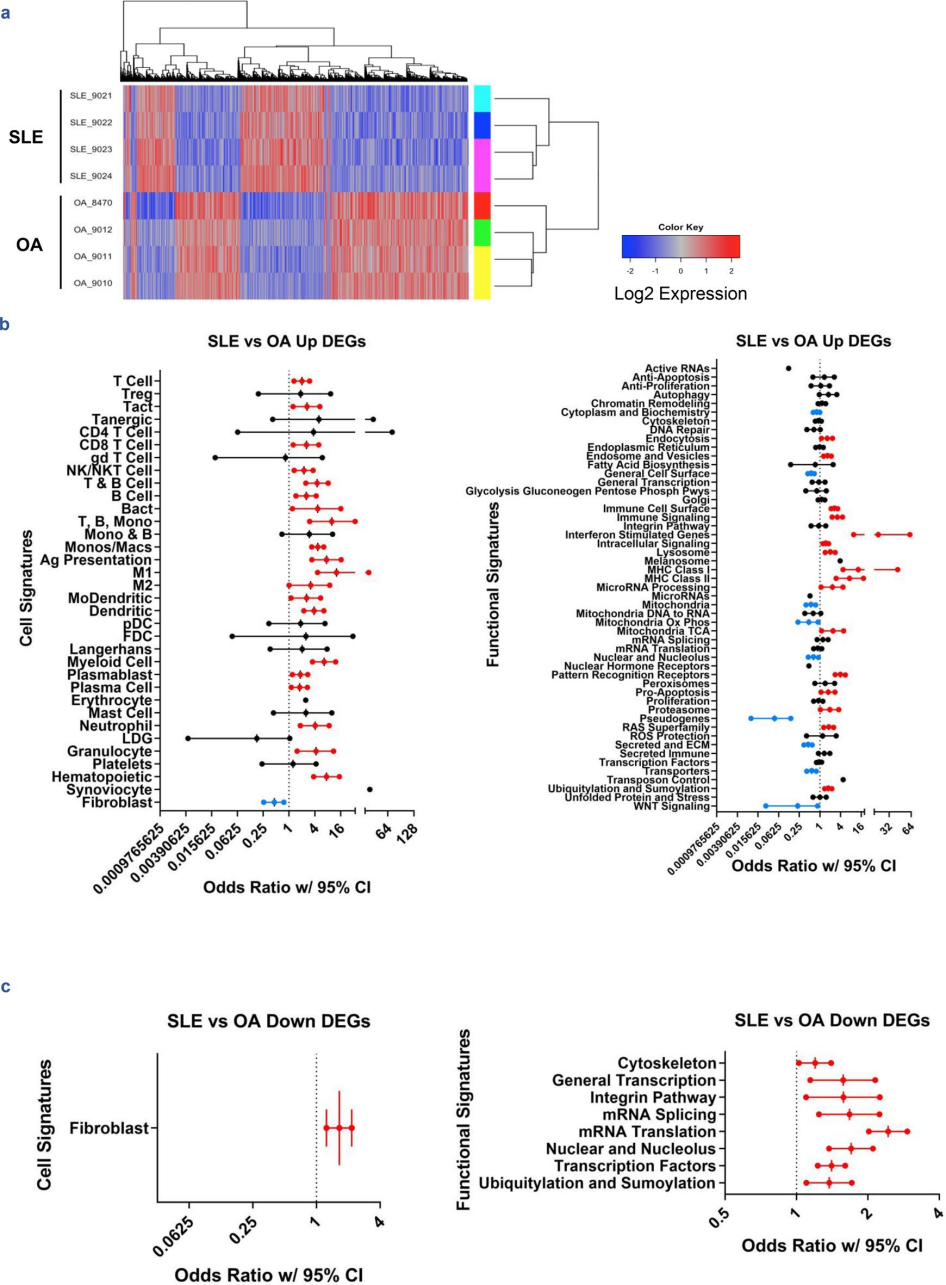
Overview of gene expression in SLE vs OA synovium. (**a**) Heatmap of 6496 DEGs from LIMMA analysis of SLE and OA synovial gene expression data generated using the R suite and Bioconductor package gplots 3.0.3 (https://CRAN.R-project.org/package=gplots). Increased (**b**) and decreased (**c**) transcripts were each characterized by cellular signatures for prevalence of specific cell types. DE transcripts were also characterized for functional signatures. Enrichment plots in (**b**,**c**) represent odds ratios bound by 95% confidence intervals (CI) using Fisher’s Exact Test. Significant enrichment by p-value (p < 0.05) and confidence intervals that exclude odds ratio = 1 are colored red and blue for positive or negative association with the sample, respectively. The x-axes are plotted on log2 scales. For categories represented by a single point, odds ratio = 0 and the data point shown represents the upper bound of the confidence interval.

Gene expression data was collected from the affected (i.e., swollen) knees of 4 female LA and 4 female OA patients by needle arthroscopy with active articular and systemic disease at the time of biopsy. Patients had not received immunosuppressive therapy or disease-modifying antirheumatic drugs before tissue sampling (see Supplementary Table S1 online for complete patient data). RNA was extracted from stored synovial samples and hybridized to Affymetrix Human Genome U133 Plus 2.0 microarrays from which differential gene expression (DE) analysis was conducted (see “Methods”). DE analysis demonstrated 6496 differentially expressed genes (DEGs) in LA versus OA (Fig. 1a), of which 2477 transcripts were upregulated and 4019 transcripts were downregulated. The upregulated DEGs included 243 immune cell-specific transcripts (odds ratio of 2.84, p < 2.2e−16, Fisher’s Exact Test), indicating a significant immune/inflammatory cell infiltrate. There was considerable enrichment of T-cell, B-cell, plasma-cell, and myeloid-cell transcripts among the upregulated DEGs (Fig. 1b), whereas fibroblast-associated genes were increased in OA. Functional enrichment analysis further indicated immune involvement via upregulated cell surface markers and immune signaling signatures. In particular, innate immune processes were enriched in LA, including interferon stimulated genes, pattern recognition receptors (PRRs), and MHC Class I and II. A number of processes related to cellular uptake and processing/packaging material inside cells were also enriched along with apoptotic pathways and the proteasome.

Of the 4019 downregulated DEGs, only 17 were immune cell transcripts and thus downregulated genes did not reflect a change in hematopoietic cell composition (odds ratio of 0.0749, p = 1). Notably, however, the fibroblast gene signature was downregulated (Fig. 1c). Functional analysis identified several molecular processes that were decreased in LA, most of which related to transcriptional activity/nuclear processes and cytoskeletal and integrin pathway changes.

Preliminary bioinformatic analysis by examination of DEGs validated previously reported findings that interferon-inducible (IFI) genes are upregulated in LA and transcripts comprising the extracellular matrix (ECM) are downregulated^9^. Our analysis expanded these insights to reveal downregulation of fibroblast-associated genes and increased expression of transcripts attributed to specific innate and adaptive immune cell types and processes. A list of genes significantly up- and downregulated in LA can be found in Supplementary Data S1 online.

**Figure 2:**
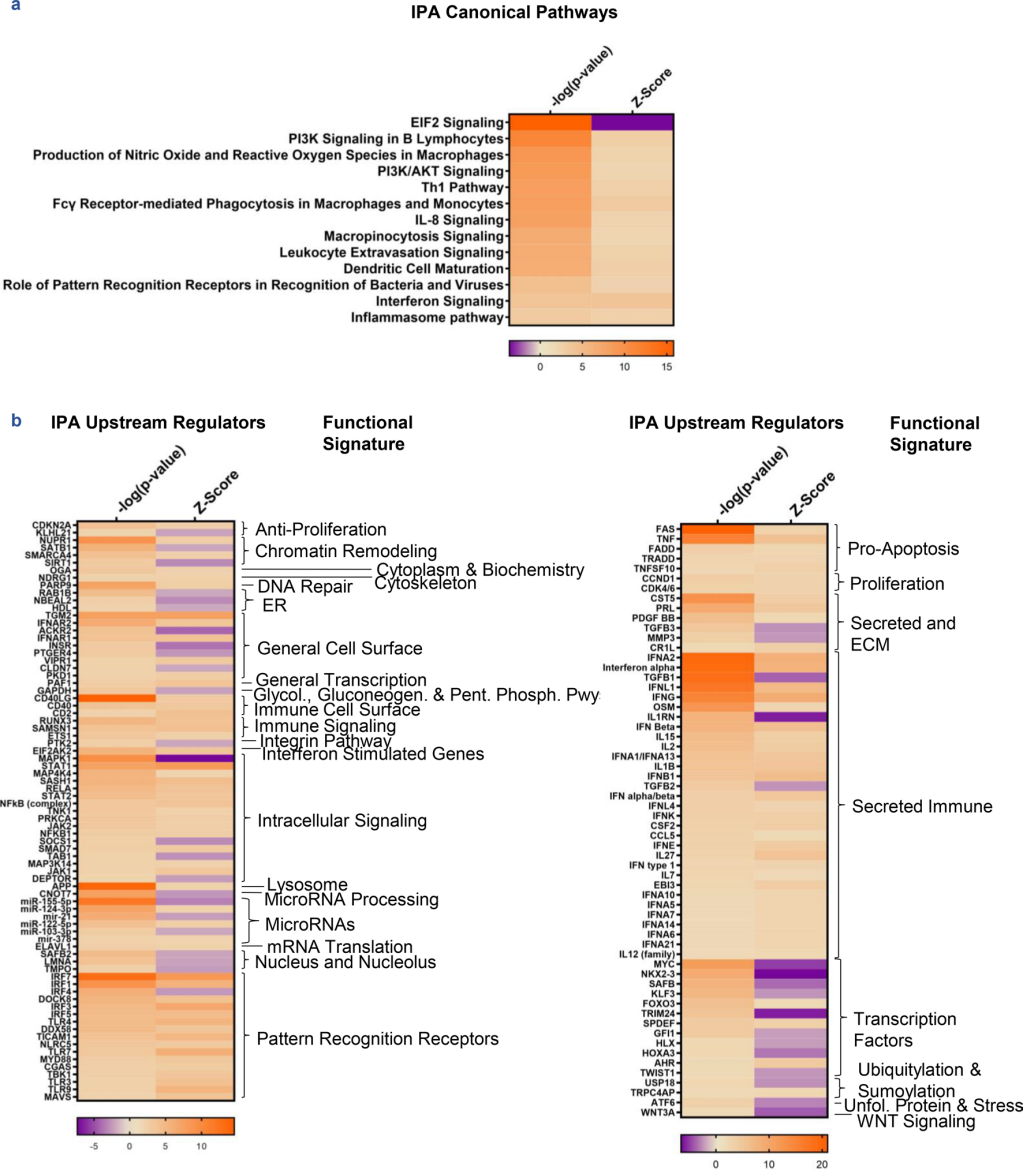
Pathway analysis of LA vs. OA gene expression. (**a**) Canonical pathways predicted by IPA based on DEGs, ordered by significance. (**b**) Significant upstream regulators predicted by IPA based on DEGs, ordered alphabetically by functional category. All canonical pathways and upstream regulators are significant by |Activation Z-Score| ≥ 2 and overlap p-value < 0.01.

We next employed Ingenuity Pathway Analysis (IPA) on LA and OA DEGs and identified predominantly innate immune signaling processes, including a proinflammatory macrophage response, interferon signaling and inflammasome pathway activation (Fig. 2a). These were confirmed by functional analysis of IPA-predicted upstream regulators (UPRs) of the disordered gene expression profiles in LA, which identified specific intracellular signaling molecules, PRRs, and secreted immune proteins, including type 1, 2, and 3 interferons (Fig. 2b). Of note, a number of pro-apoptotic genes, including *TNF*, *TNFSF10*, and *FAS* were predicted UPRs. IPA served to inform specific pathways involved in LA that appear to primarily involve myeloid cells as well as specific molecules driving disease that may be able to be targeted with specific therapies. The full export of IPA canonical pathway and upstream regulator data can be found in Supplementary Data S3–6 online.

To confirm DEG-identified molecular pathways and further interrogate LA gene expression, we implemented weighted gene co-expression network analysis (WGCNA), which serves as an orthogonal bioinformatic approach. WGCNA identified 52 modules, six of which were highly co-expressed and significantly associated with LA (see Supplementary Figs. S1, S2, and Supplementary Table S2 online). Of the six modules with significant, positive correlations to features of lupus, some correlated with the presence of LA and some modules correlated with SLE disease activity index (SLEDAI) and anti-dsDNA. The associations of the WGCNA modules with features of LA can be seen in Supplementary Figs. S1, S3, S4, and Supplementary Data S7–13 online. Many of the modules contained immune/inflammatory cell signatures. Notably, plasma cell genes were found in the midnightblue module and included IgG1, IgM, and IgD, indicating both pre- and post-switch plasmablasts/plasma cells as well as the presence of Igκ, Igλ, and numerous light (VL) chains, signifying a polyclonal population (see Supplementary Fig. S5 online). Midnightblue, however, was upregulated in only two of four lupus patients and negatively correlated with systemic disease (see Supplementary Fig. S1 online). The blue module, which was negatively correlated with LA, was enriched in synovial fibroblasts (see Supplementary Fig. S4, Supplementary Table S3 online), whereas the brown module, which was positively associated with LA, was not enriched for synovial fibroblasts (see Supplementary Fig. S3 online). These associations indicate that a polyclonal plasma cell population may be present in a subset of LA patients and that the fibroblast population is significantly altered in LA compared to OA.

WGCNA modules were enriched in immune/inflammatory processes, most of which overlapped with DEG-defined functional enrichment, and additionally included enrichment of autophagy pathways and mRNA splicing (see Supplementary Fig. S6 online). Further IPA canonical pathway analysis, IPA UPR analysis, and clustering of WGCNA modules based on protein–protein interactions (see Supplementary Fig. S7 online) indicated that the brown and navajowhite2 modules were characterized by myeloid cell responses, whereas the honeydew1 module was most characterized by interferon signaling. The darkgrey module was characterized by cellular activation, antigen presentation, and proinflammatory signaling. In contrast, the midnightblue module revealed T-cell: B-cell crosstalk, T-cell activation and differentiation, and B-cell signaling. Finally, salmon4 was not enriched in immune cells (see Supplementary Fig. S3 online). Functional and pathway analysis of these LA-associated modules suggest that co-expressed genes in LA act in a manner consistent with myeloid-mediated effector phase responses, and that involvement of T-cells, B-cells, and plasma cells is present, but less robust.

### Lymphocyte trafficking and germinal center (GC) activity in LA

Next, after establishing the presence of immune cells and inflammatory signaling in LA, we assessed chemokine receptor–ligand pairs and adhesion molecules to understand mechanisms of immune/inflammatory cell localization in LA. Numerous chemokine receptor–ligand pairs were expressed in SLE synovium, including *CCR5–CCL4/5/8*, *CCR1–CCL5/7/8/23,* and *CXCR6–CXCL16* (Table 1). Of note, *CXCL13* was expressed in the midnightblue module and upregulated, although its receptor *CXCR5* was not detected in LA-associated WGCNA modules nor in DEGs. Adhesion molecules were found in LA-associated WGCNA modules including *VCAM1*, *CD44*, *CADM3*, and *ITGB2*.

**Table 1:** Chemokine receptor–ligand pairs and adhesion molecules associated with LA.

Gene transcript	Name	SLE vs OA analysis	
		DE LFC	WGCNA LA-associated module
CCL19	Chemokine (C–C motif) ligand 19	n/s	Honeydew1
*CCR2*	*Chemokine *(*C–C motif*) *receptor 2*	*1.50*	*Midnightblue*
*CCL2*	*Chemokine *(*C–C motif*)* ligand 2*	*n/s*	*Darkgrey*
*CCL7*	*Chemokine *(*C–C motif*)* ligand 7*	*n/s*	*Darkgrey*
*CCL8*	*Chemokine *(*C–C motif*)* ligand 8*	*2.76*	*Darkgrey*
CCR5	Chemokine (C–C motif) receptor 5	1.90	Navajowhite2
CCL4	Chemokine (C–C motif) ligand 4	2.67	Brown
CCL5	Chemokine (C–C motif) ligand 5	1.94	Midnightblue
CCL8	Chemokine (C–C motif) ligand 8	2.76	Darkgrey
*CCR1*	*Chemokine *(*C–C motif*)* receptor 1*	*2.14*	*Brown*
*CCL5*	*Chemokine *(*C–C motif*)* ligand 5*	*1.94*	*Midnightblue*
*CCL7*	*Chemokine *(*C–C motif*)* ligand 7*	*n/s*	*Darkgrey*
*CCL8*	*Chemokine *(*C–C motif*)* ligand 8*	*2.76*	*Darkgrey*
*CCL23*	*Chemokine *(*C–C motif*)* ligand 23*	*0.670*	
CCR3	Chemokine (C–C motif) receptor 3	n/s	Darkgrey
CCL5	Chemokine (C–C motif) ligand 5	1.94	Midnightblue
CCL7	Chemokine (C–C motif) ligand 7	n/s	Darkgrey
CCL8	Chemokine (C–C motif) ligand 8	2.76	Darkgrey
*CCRL2*	*Chemokine *(*C–C motif*)* receptor-like 2*	*1.04*	*Navajowhite2*
CKLF	Chemokine like factor	0.297	
*CMKLR1*	*Chemokine-like receptor 1*	*1.59*	*Darkgrey, Honeydew1*
CXCL2	Chemokine (C–X–C motif) ligand 2	2.98	Honeydew1
CXCL3	Chemokine (C–X–C motif) ligand 3	1.77	
CXCL8	Chemokine (C–X–C motif) ligand 8	2.17	Brown, Darkgrey
*CXCR3*	*Chemokine *(*C–X–C motif*)* receptor 3*	*1.45*	*Midnightblue*
*CXCL9*	*Chemokine *(*C–X–C motif*)* ligand 9*	*5.59*	*Midnightblue*
*CXCL10*	*Chemokine *(*C–X–C motif*)* ligand 10*	*4.81*	*Midnightblue*
*CXCL11*	*Chemokine *(*C–X–C motif*)* ligand 11*	*3.32*	*Midnightblue*
CXCR4	Chemokine (C–X–C motif) receptor 4	1.39	Brown
*CXCL13*	*Chemokine *(*C–X–C motif*)* ligand 13*	*3.47*	*Midnightblue*
CXCR6	Chemokine (C–X–C motif) receptor 6	n/s	Midnightblue
CXCL16	Chemokine (C–X–C motif) ligand 16	0.768	Navajowhite2
*CXCL11*	*Chemokine *(*C–X–C motif*)* ligand 11*	*3.32*	*Midnightblue*
CX3CL1	Chemokine (C–X3–C motif) ligand 1	0.453	
*XCL1*	*Chemokine *(*X–C motif*)* ligand 1*	*n/s*	*Midnightblue*
ALCAM	Activated leukocyte cell adhesion molecule	1.55	
VCAM1	Vascular cell adhesion molecule 1	n/s	Navajowhite2
CD44	CD44 molecule	1.25	Brown, Darkgrey
ITGB1	Integrin subunit beta 1	− 0.255	
ITGB2	Integrin subunit beta 2	1.56	Brown, Honeydew1
ICAM1	Intercellular adhesion molecule 1	0.861	Darkgrey, Honeydew1, Midnightblue
ICAM3	Intercellular adhesion molecule 3	n/s	Midnightblue
PECAM1	Platelet/endothelial cell adhesion molecule 1	0.618	Salmon4
SDK1	Sidekick cell adhesion molecule 1	− 0.892	
SDK2	Sidekick cell adhesion molecule 2	− 1.33	
CADM1	Cell adhesion molecule 1	− 0.974	
CADM3	Cell adhesion molecule 3	n/s	Darkgrey
JAM2	Junctional adhesion molecule 2	− 0.587	
JAM3	Junctional adhesion molecule 3	− 0.673	
MCAM	Melanoma cell adhesion molecule	− 1.08	

DEGs and LA-associated WGCNA modules were assessed for adhesion molecules and chemokine receptor–ligand pairs. Receptor–ligand pairs are grouped together in the table with groupings alternately italicised. Log fold changes rounded to 3 significant figures are presented where available; otherwise, *n/s *not significant.

We also examined expression of specific follicular helper T cell (T_fh_) and GC B-cell markers to determine whether GCs might contribute to LA pathogenesis (see Supplementary Fig. S8 online). *ICOS*, but not other T_fh_ markers, were found in LA. In addition, several GC B-cell markers were upregulated in SLE synovium, including, *CXCL13* and *IRF4*. However, *BCL6* and *RGS16* were notably downregulated and *RGS13* was not differentially expressed between SLE and OA. A cluster of GC B-cell markers that tended to be upregulated were co-expressed in the midnightblue module, which contained a lymphocyte signal. These analyses indicate that while there may be GC-like activity in some LA patients, fully-formed GCs are not a likely feature of lupus synovitis and most immune/inflammatory cells probably migrate into the tissue by chemokine signaling.

### Gene set variation analysis (GSVA) enrichment of immune and tissue populations and signaling pathways

**Figure 3:**
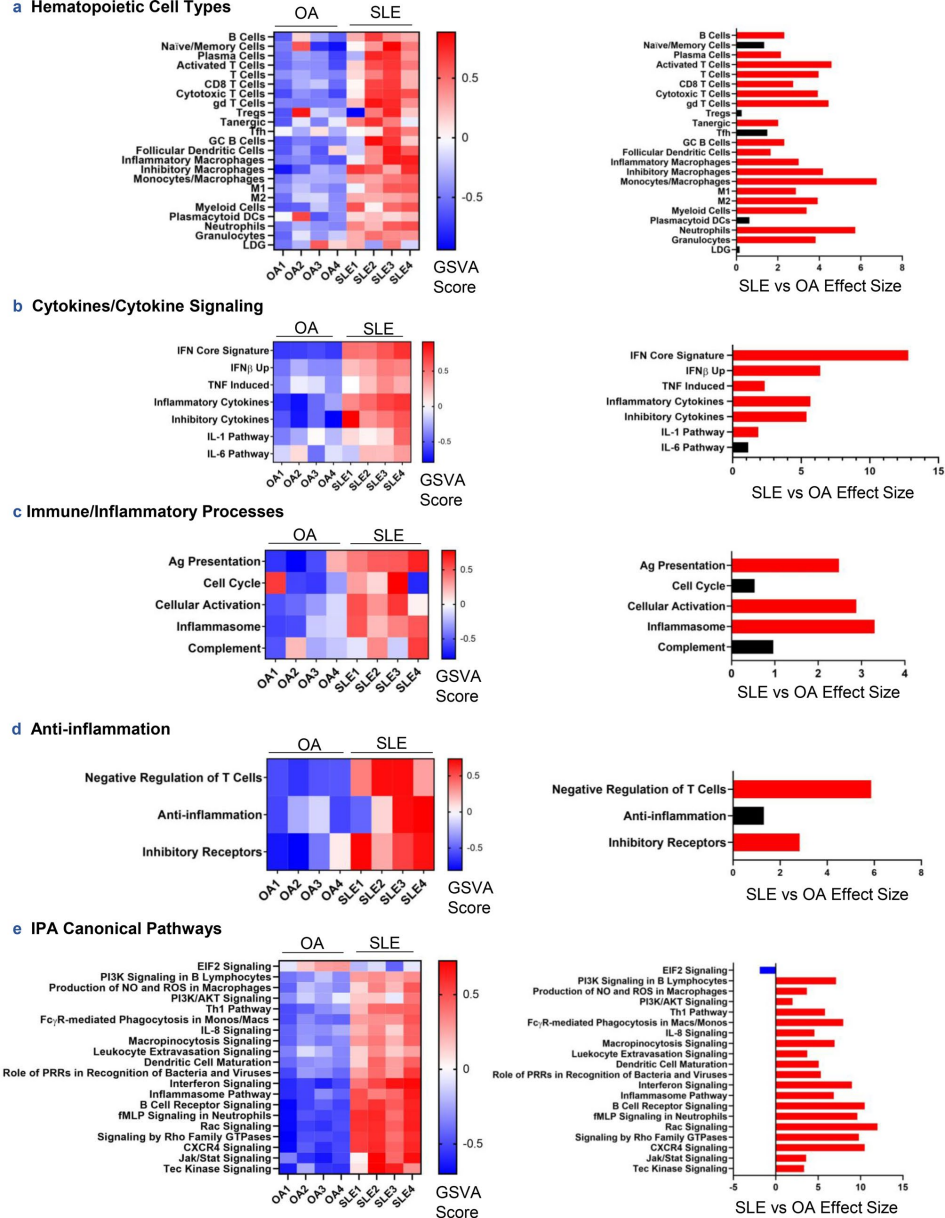
GSVA of hematopoietic cell types (**a**), cytokine signatures and signaling pathways (**b**), immune/inflammatory processes (**c**), anti-inflammatory processes (**d**), and IPA-predicted canonical signaling pathways from DEGs in SLE vs OA synovium (**e**) was conducted on log2-normalized gene expression values from OA and SLE synovium. Hedge’s g effect sizes were calculated with correction for small sample size for each gene set and significant differences in enrichment between cohorts were found by Welch’s *t* test (p < 0.05), shown in the panels on the right. Red and blue effect size bars represent significant enrichment in SLE and OA, respectively.

To assess the differences between SLE and OA synovitis on an individual sample basis, GSVA of various informative gene sets was carried out (Fig. 3, see Supplementary Data S14 online). Enrichment of hematopoietic cell types confirmed the presence of an immune infiltrate in LA, but not OA (Fig. 3a). Most cell types, including lymphoid and myeloid populations, were enriched. Cytokine signaling was also enriched in LA, including both proinflammatory cytokine signaling as well as inhibitory cytokines (Fig. 3b). Of note, the downstream signature induced by TNF signaling was significantly enriched in LA (p = 0.00918). Whereas antigen presentation markers, cellular activation markers, and the inflammasome pathway were enriched in LA compared to OA, a cell cycle/proliferation signature and complement pathways were not significantly enriched (Fig. 3c; p = 0.420 and p = 0.169, respectively). Along with upregulation of inhibitory cytokines, inhibitory receptors and negative regulation of T cells were enriched in LA (Fig. 3d). Most of the previously noted IPA-predicted canonical signaling pathways were enriched in SLE synovium aside from signaling by the eukaryotic initiation factor eIF2 (Fig. 3e). Our initial GSVA results validated findings from DE analysis and WGCNA on a per sample basis and implicate broad immune activation in LA.

**Figure 4:**
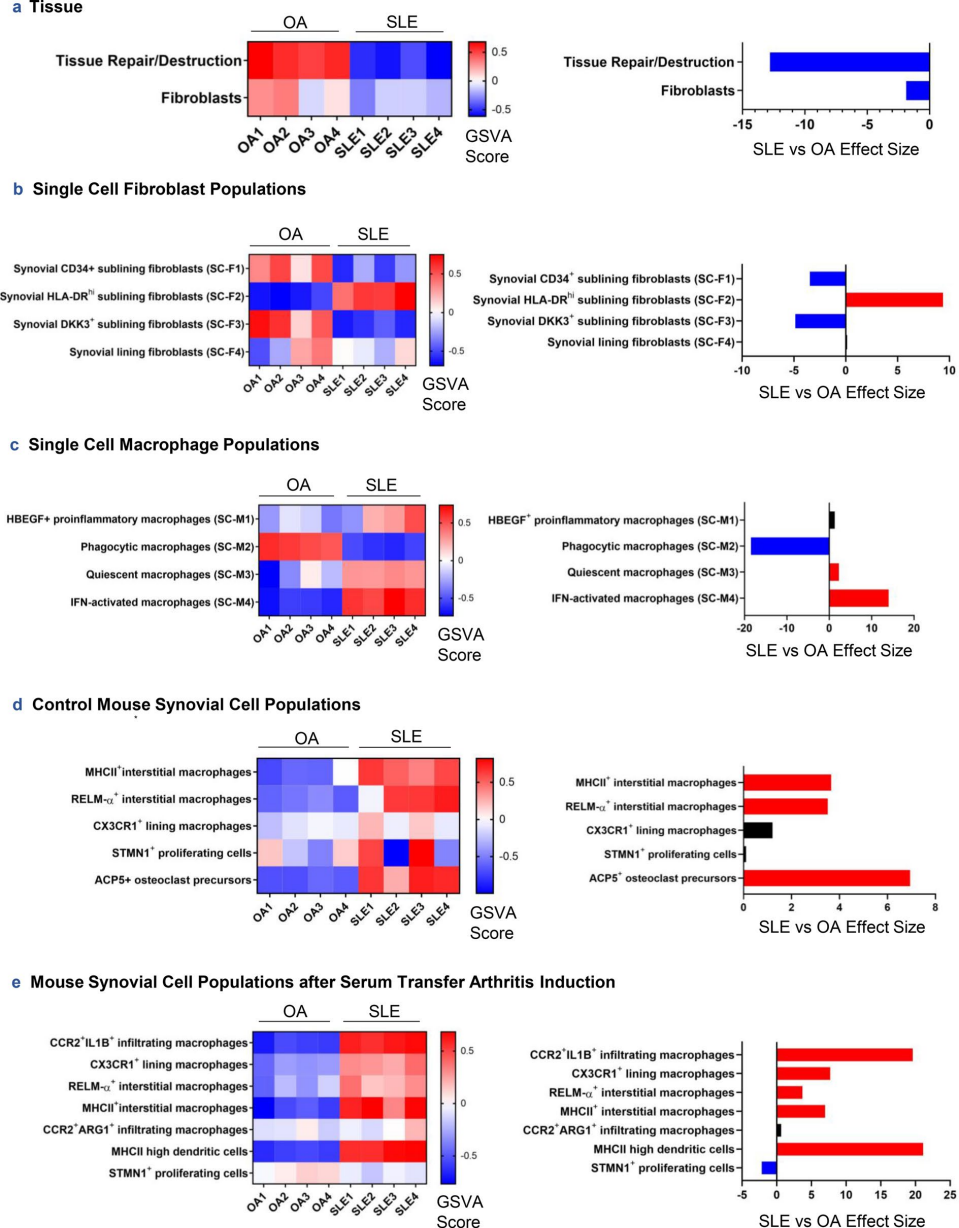
GSVA of synovial tissue processes and specific cell types (**a**) and recently published synovium-specific cell subtypes in human RA, OA, and mouse synovium (**b**–**e**) was conducted on log2-normalized gene expression values from OA and SLE synovium. Hedge’s g effect sizes were calculated with correction for small sample size for each gene set and significant differences in enrichment between cohorts were found by Welch’s *t* test (p < 0.05), shown in the panels on the right. Red and blue effect size bars represent significant enrichment in SLE or OA, respectively. Literature-derived signatures in (**b**–**e**) underwent co-expression analyses before being used as GSVA gene sets (see “Methods”).

Conversely, OA synovium was enriched in tissue repair/destruction and markers of fibroblasts (Fig. 4a). Querying LA and OA synovial expression profiles with the co-expressed genes from fibroblast subpopulations described in human RA and OA synovium^10^, we found that two resident synovial sublining fibroblast populations, *CD34*^+^ and *DKK3*^+^, were significantly increased in the OA samples over LA (Fig. 4b; p = 0.00148 and 0.00213, respectively). However, co-expressed genes characterizing the *HLA-DR*^*hi*^ sublining fibroblast population were significantly enriched in LA (p = 2.70e−05). Interestingly, using the same approach to assess macrophage populations also described in human RA and OA^10,11^ we found quiescent macrophages and interferon (IFN)-activated macrophages significantly increased in LA (Fig. 4c; p = 0.0332 and 1.14e−06, respectively), whereas phagocytic macrophages were associated with OA. *HBEGF*^+^ proinflammatory macrophages tended to be enriched in LA but did not reach statistical significance (p = 0.0993).

Given the overlap of features from specific macrophage and fibroblast populations between human RA and LA or OA, we thought it pertinent to determine whether LA macrophages additionally share features with other unique macrophage subpopulations described in mouse synovium, including a *CX3CR1*^+^ resident subtype resembling epithelial cells^12^. We compared signatures of sorted mouse synovial CD45^+^CD11b^+^Ly6G^−^ mononuclear phagocytes using marker genes from single-cell RNA sequencing (scRNA-seq) clusters and examined enrichment of these populations characteristic of healthy control mice and an inflammatory murine arthritis model. Taking the co-expressed genes of the human orthologs of the macrophage signatures detected in healthy mouse synovium, we found that two types of interstitial macrophages, including *MHCII*^+^ and *RELM-α*^+^ populations, were significantly more abundant in LA (p = 0.00549 and 0.00802, respectively) as well as a group of *ACP5*^+^ osteoclast precursors (Fig. 4d; p = 9.37e−04). The *CX3CR1*^+^ lining macrophage population from healthy mice, reported to be protective by forming a barrier around the joint cavity^12^, was not significantly enriched in either LA or OA synovium (p = 0.110); however, the *CX3CR1*^+^ lining macrophage population from murine inflammatory arthritis was increased in LA (Fig. 4e). Additionally, inflammatory arthritis-associated *MHCII*^+^ and *RELM-α*^+^ interstitial macrophages were enriched in LA as were *MHCII*^*hi*^ dendritic cells and *CCR2*^+^*IL1B*^+^ monocyte-derived macrophages. Thus, macrophage subpopulations identified in LA include those that share features with resident interstitial populations and inflammatory, monocyte-derived populations in murine arthritis.

### Comparison of gene expression in LA and RA synovitis

**Figure 5:**
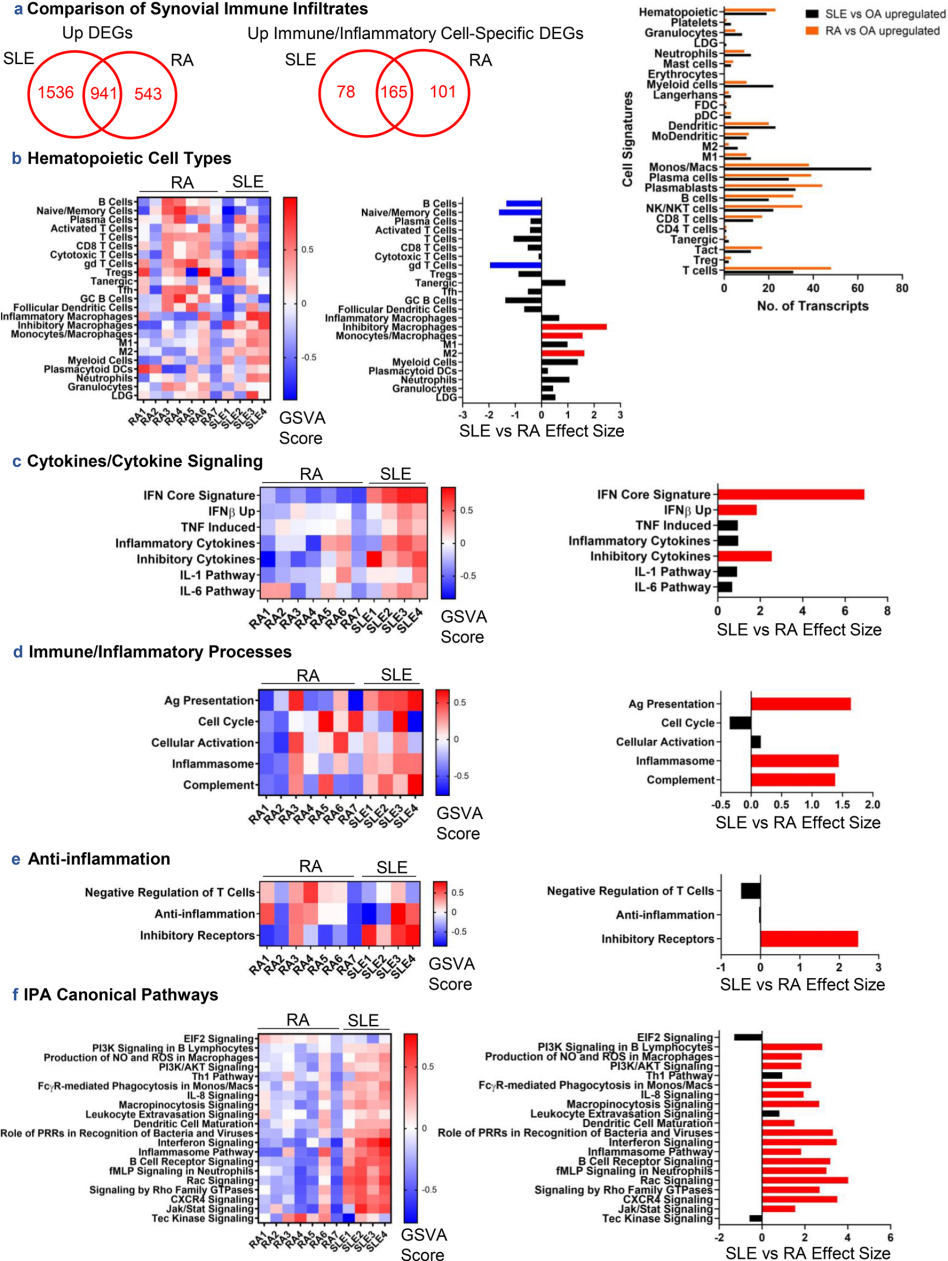
A comparison of immune/inflammatory gene signatures between SLE and RA synovium using 7 RA patients from GSE36700. (**a**) Upregulated DEGs were identified between RA and OA synovium, compared to DEGs from SLE vs OA synovium, and characterized by cellular signatures. GSVA of hematopoietic cell types (**b**), cytokine signatures and signaling pathways (**c**), immune/inflammatory processes (**d**), anti-inflammatory processes (**e**), and IPA-predicted canonical signaling pathways from DEGs in SLE vs OA synovium (**f**) was conducted on log2-normalized gene expression values from SLE and RA synovium. Hedge’s g effect sizes were calculated with correction for small sample size for each gene set and significant differences in enrichment between cohorts were found by Welch’s *t* test (p < 0.05), shown in the panels on the right. Red and blue effect size bars represent significant enrichment in SLE or RA, respectively.

To fine-tune our characterization of LA, we also compared gene expression data against human inflammatory arthritis. Using seven RA samples as comparators (see Supplementary Table S1 online), we observed fewer genes upregulated in RA; however, 18% of these genes identified immune/inflammatory cells compared with 10% of genes upregulated in LA (Fig. 5a). Characterization of upregulated DEGs by cell signatures revealed greater numbers of myeloid and monocyte/macrophage-specific transcripts in LA compared to RA, whereas immune infiltrates in RA were more characteristic of T- and B-cells. B-cells, naïve/memory cells, and gamma delta (gd) T cells were significantly increased in RA over LA (Fig. 5b; p = 0.0249, p = 0.0121, and p = 0.00689, respectively), whereas monocytes/macrophages, inhibitory macrophages, and M2 macrophages were significantly increased in LA over RA (p = 0.0160, p = 0.00306, and p = 0.00719, respectively).

Components of the effector phase including interferon, inflammasome, and complement pathways were substantially enriched in LA, whereas negative regulation of T-cells and cell cycle tended to be enriched in RA (Fig. 5c–e). Downstream signatures of TNF, IL-1, and IL-6 tended to be enriched in LA compared to RA. Finally, other pathways involved in response to stimuli, phagocytosis, chemokine signaling, B-cell receptor signaling, and PI3K signaling, were considerably enriched in LA (Fig. 5f).

Given the observed alterations in fibroblast and macrophage compartments in LA compared to OA, we also examined these populations in RA versus LA. Transcripts associated with tissue repair/destruction were significantly enriched in RA (see Supplementary Fig. S9 online; p = 9.95e−04), but enrichment of general fibroblast markers was not uniform in either tissue. Nonetheless, *HLA-DR*^*hi*^ sublining and *CD55*^+^ lining fibroblasts were significantly associated with LA (p = 0.00247 and p = 1.39e−04, respectively), whereas the *CD34*^+^ and *DKK3*^+^ sublining populations were depleted in LA compared to RA.

*HBEGF*^+^ proinflammatory macrophages and phagocytic macrophages tended to be more enriched in RA patients, although not uniformly, whereas quiescent and IFN-activated macrophages were more enriched in LA, the latter population reaching statistical significance (see Supplementary Fig. S9 online; p = 0.00857)^10,11^. Interestingly, although serum transfer in K/BxN mice has been used to model RA^13^, when detected, the normal and arthritis murine equivalent macrophage populations trended towards enrichment in LA, including the *CX3CR1*^+^ lining macrophages^12^.

### Compounds predicted to target LA

**Figure 6:**
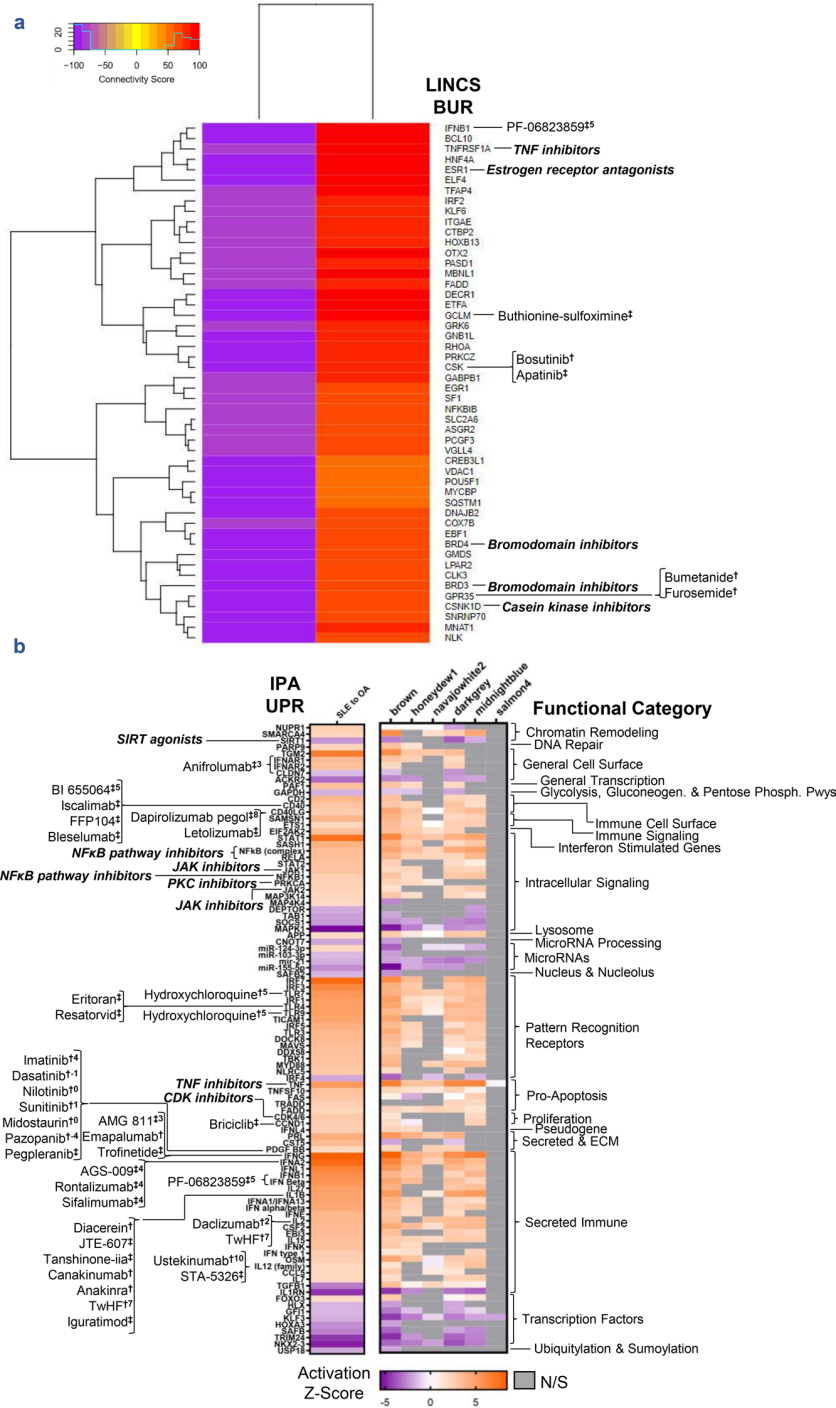
LINCS biological upstream regulators and IPA upstream regulators operative in LA are potential druggable targets. (**a**) The top 50 targets (BURs) opposing the LA gene signature from LINCS knock down (KD) and overexpression (OE) assays summarized by connectivity score and matched to appropriate targeting drugs. KD and OE data were filtered for connectivity scores in the [− 75 to − 100] and [50 to 100] ranges, respectively. The heatmap was generated using the R suite and Bioconductor package gplots 3.0.3 (https://CRAN.R-project.org/package=gplots). (**b**) The consensus IPA-predicted UPRs between DEGs and LA-associated WGCNA modules summarized by Activation Z-Score, functional category, and also matched to appropriate targeting drugs. Drugs and compounds targeting the BURs and UPRs were sourced from LINCS/Connectivity Map-Linked User Environment (CLUE), IPA, literature mining, CoLTS^45^, STITCH, and clinical trials databases. Drug annotations are grouped together by target and CoLTS scores (range − 16 to + 11) are displayed as integers in superscript. Some upstream regulators are matched to groups of drugs (e.g., NFκB pathway inhibitors, bold, italicized), for which the full list of drug–target matches can be found in Supplementary Data S15–16 online. ^P^Preclinical; ^‡^drug in development/clinical trials; ^†^FDA-approved.

Finally, in addition to analysis of mechanisms involved in LA pathogenesis, we aimed to suggest drugs and compounds that could prove useful in the specific treatment of arthritis in lupus patients. Drugs predicted to reverse the abnormal gene expression profile of LA were identified by connectivity mapping to the Library of Integrated Network-Based Cellular signatures (LINCS) database (see “Methods”) and are shown in Table 2. Most abundantly predicted compounds include anti-cancer drugs targeting tubulin polymerization, MAPK signaling, and EGFR signaling, as well as current lupus standard-of-care therapies, including corticosteroids and prostaglandin synthesis inhibitors. Interestingly, a few alternative medicines were predicted to counteract LA, including capsaicin, resveratrol, and caffeine. In addition to the LINCS-predicted compounds, we sought to expand the potential list of therapies to include those targeting biological upstream regulators (BURs). The top 50 BURs determined by connectivity scoring with gene expression generated by knock down or overexpression studies in cell lines are summarized in Fig. 6a along with drugs that could potentially directly target these BURs, including TNF, type 1 IFN, bromodomain and casein kinase inhibitors. Finally, the UPRs predicted by IPA were also matched with potential targeting drugs (Fig. 6b). Notably, 26% of drugs targeting IPA upstream regulators were also predicted by LINCS BURs drug–target matches (see Supplementary Data S15–16 online), and included inhibitors of TNF, type I interferon, the NFκB pathway, JAK, and CDK.

**Table 2:** Compounds targeting LA.

Target	Count	Range	Mean ± SEM	Top LINCS drug	Related drug
PKC	2	(− 97.13)–(− 99.70)	− 98.41 ± 1.28	Enzastaurin^‡^	Midostaurin^†1^
GSK3	5	(− 81.19)–(− 99.96)	− 95.05 ± 3.56	SB-216763^P^	Enzastaurin^‡^
RAF	3	(− 89.13)–(− 98.35)	− 94.91 ± 2.91	Vemurafenib^†−6^	Sorafenib^†−3^
CDK	4	(− 81.19)–(− 99.96)	− 92.59 ± 4.49	SB-216763^P^	Palbociclib^†3^
GR agonist	11	(− 83.48)–(− 97.95)	− 91.61 ± 1.53	Dexamethasone^†^	Prednisone^†^
ROCK1/2	3	(− 90.80)–(− 91.72)	− 91.15 ± 0.288	Fasudil^‡^	KD025^†7^
Cholinesterase	2	(− 88.16)–(− 93.36)	− 90.76 ± 2.60	Mestinon^†^	Isoflurophate^†^
Retinoid R agonist	4	(− 81.80)–(− 95.44)	− 90.76 ± 3.05	TTNPB^P^	Acitretin^†^
VEGFR	2	(− 83.38)–(− 97.26)	− 90.32 ± 6.94	Sorafenib^†−3^	Sunitinib^†0^
MAP2K1/2	6	(− 80.48)–(− 98.40)	− 90.17 ± 2.64	Selumetinib^†^	Vemurafenib^†−6^
MAPK	4	(− 86.79)–(− 95.39)	− 90.00 ± 1.92	FR-180204^P^	Losmapimod^‡^
mTORC1/2	2	(− 88.13)–(− 91.07)	− 89.60 ± 1.47	Sirolimus^†−2^	*N*-acetyl cysteine^†3^
EGFR	6	(− 79.42)–(− 99.14)	− 89.58 ± 3.13	Lapatinib^†0^	Gefitinib^†1^
Tyrosine kinase	3	(− 81.70)–(− 97.26)	− 89.49 ± 4.49	Sorafenib^†−3^	Nilotinib^†0^
Tubulin	14	(− 82.65)–(− 96.56)	− 88.98 ± 1.24	Epothilone^‡^	Albendazole^†^
b2 adrenergic R agonist	3	(− 82.19)–(− 90.15)	− 88.82 ± 2.58	Isoxsuprine^‡^	Albuterol^†^
5 alpha reductase	2	(− 86.29)–(− 91.18)	− 88.73 ± 2.44	Alpha-estradiol^P^	Acexamic acid^†^
TRPV agonist	2	(− 80.20)–(− 97.26)	− 88.73 ± 8.53	Capsaicin^†^	Evodiamine
PARP	6	(− 77.24)–(− 98.35)	− 88.28 ± 3.17	Rucaparib^†^	Niraparib^†3^
Angiotensin R	2	(− 84.35)–(− 92.20)	− 88.28 ± 3.93	Candesartan^†^	Azilsartan^†^
P450	3	(− 81.70)–(− 92.02)	− 87.53 ± 3.06	Proadifen^P^	Resveratrol^†4^
Androgen R	5	(− 81.04)–(− 96.04)	− 87.36 ± 2.70	BMS-641988^‡^	Apalutamide^†^
Na channel	9	(− 79.66)–(− 98.23)	− 87.05 ± 1.95	Phenamil^P^	Benzocaine^†^
HIV protease	2	(− 86.19)–(− 87.78)	− 86.98 ± 0.79	Lopinavir^†^	Nelfinavir^†2^
TGFBR	3	(− 80.73)–(− 98.13)	− 86.93 ± 5.61	SB-525334	Pirfenidone^†^
PI3K (pan)	2	(− 84.70)–(− 88.90)	− 86.80 ± 2.10	PIK-90^P^	Idelalisib^†1^
HMG-CoA reductase	4	(− 76.32)–(− 93.09)	− 86.47 ± 3.62	Atorvastatin^†3^	Rosuvastatin^†3^
PRKDC	3	(− 83.42)–(− 90.00)	− 86.04 ± 2.02	NU-7026^P^	Caffeine
PDE	6	(− 77.14)–(− 95.12)	− 85.63 ± 3.29	Bucladesine^P^	Dipyridamole^†4^
MDM	2	(− 75.96)–(− 94.33)	− 85.15 ± 9.18	Serdemetan^‡^	Idasanutlin^‡^
NSAID/prostaglandin	9	(− 76.19)–(− 95.07)	− 84.93 ± 1.97	SC-560^P^	Aspirin^†^
HDAC	2	(− 79.79)–(− 90.05)	− 84.92 ± 5.13	Valproic acid^†2^	Vorinostat^†6^
ACE	2	(− 81.90)–(− 86.18)	− 84.04 ± 2.14	Enalapril^†^	Alacepril^†^
DHFR	2	(− 75.51)–(− 92.42)	− 83.96 ± 8.45	Pyrimethamine^†^	Methotrexate^†1^
AMPA R agonist	2	(− 79.16)–(− 84.63)	− 81.90 ± 2.74	Nobiletin	Aniracetam^†^
Topoisomerase II	3	(− 77.89)–(− 88.85)	− 81.87 ± 3.50	Razoxane^‡^	Doxorubicin^†^
IGF1R	2	(− 78.32)–(− 83.82)	− 81.07 ± 2.75	GSK-1904529A^P^	Ceritinib^†−4^
HSP90AA1	2	(− 76.84)–(− 85.28)	− 81.06 ± 4.22	Gedunin^P^	Rifabutin^†^
NAMPT	2	(− 76.91)–(− 84.58)	− 80.75 ± 3.83	FK-866^‡^	GMX-1778^‡^
Calcineurin	2	(− 79.03)–(− 80.44)	− 79.73 ± 0.70	Cyclosporine^†−5^	Tacrolimus^†5^
DNMT	2	(− 75.19)–(− 83.44)	− 79.31 ± 4.12	Decitabine^†^	Azacitidine^†^
Carbonic anhydrase	2	(− 78.54)–(− 78.81)	− 78.67 ± 0.13	Chlortalidone^†^	Acetazolamide^†^

Compounds predicted by LINCS to oppose the LA gene signature were summarized by their drug targets for every target with at least two compounds. Compounds were analyzed if corresponding connectivity scores fell in the range of − 75 to − 100 to reflect most opposite gene signatures and if the connectivity of antagonists and agonists of the same target were acting in opposite directions. Top LINCS Drug represents the most negative-scoring compound for a specific target category. Related drug represents the most immunologically relevant or well-known drug for a specific target category. Where applicable, CoLTS scores (range − 16 to + 11)^45^ are displayed as integers in superscript. *R *receptor; ^P^preclinical (animal model); ^‡^drug in development/clinical trials; ^†^FDA-approved.

## Discussion

Using previously reported data, we applied multiple contemporary bioinformatic approaches to enhance current understanding of molecular signatures driving LA pathogenesis. Analyses of DEGs revealed a uniform inflammatory infiltrate in LA of mostly myeloid lineage cell types, including monocytes, M1 macrophages, antigen presenting cells, and other myeloid and hematopoietic cells. WGCNA indicated enrichment of other immune cell types, including activated and effector T-cells, NK cells, B-cells, plasma cells/plasmablasts, and both M1 and M2-polarized macrophages, suggesting both innate and adaptive mechanisms at play in LA, although the involvement of adaptive immune cells was less uniform than that of the innate immune system. Our DE analysis confirmed the previous report of an upregulated interferon signature in LA and decreased expression of ECM constituents^9^. Our analysis also confirmed the presence of T-cells, B-cells, plasma cells, and macrophages by calculating enrichment (in multiple ways) of gene sets known to be associated with specific cell types, whereas the original report indicated the presence of these immune cells by computing semiquantitative scores of singular cell markers from immunohistochemical staining. We were also able to obtain a more granular view of cell types in LA with the identification of M1 and M2 macrophages, neutrophils, granulocytes, activated T-cells, and dendritic cells, which suggests a more robust immune infiltrate than originally reported.

Our findings indicated that myeloid-lineage cells were enriched in LA and, therefore, may play a central role in the observed inflammation. IPA revealed monocyte/macrophage-mediated phagocytosis and nitric oxide and reactive oxygen species production signaling pathways, and GSVA confirmed gene expression profiles of both inflammatory M1 and inhibitory M2 macrophages in LA. This aligns with prior histology suggesting the presence of infiltrating macrophages^9^ and recent analysis of myeloid cells in SLE blood associated an M1 inflammatory phenotype with active versus inactive disease^14^. By comparison to single-cell transcriptional profiles from sorted murine synovial CD45^+^Cd11b^+^Ly6G^−^ cells^12^, significant enrichment of resident macrophage populations as well as non-resident infiltrating populations were identified, as well as anti-inflammatory macrophage subpopulations and inhibitory and inflammatory cytokines, indicating that multiple macrophage subtypes and their secreted products may contribute to and perhaps protect against LA pathogenesis.

Of particular interest in lupus pathophysiology is the contribution of interferons. The original report of this dataset noted significant upregulation of IFI genes through DE analysis confirmed by immunostaining and real-time RT-PCR^9^. Lauwerys et al. (2015) additionally compared gene expression from the same generated SLE dataset to RA, OA, psoriatic arthritis, and microcrystalline arthritis synovial gene profiles in which type I interferon-induced genes IFI27, ISG15, RAD2, IFI6, IFIT3, and OAS1 were among the top 100 discriminant genes defining SLE between the 5 arthritides^15^. We confirmed this finding in our own differential gene expression analysis pipeline and through each individual method employed herein. In addition to enrichment of the core type I interferon signature in LA, we also found ongoing signaling by type I, type II, and type III interferons, though we could not attribute interferon production to any one cell type.

Fibroblast-unique genes were downregulated in LA, possibly representing local relative loss or diminished/altered function of resident fibroblasts. Pathologic fibroblast populations, potentially contributing to local tissue damage, have been shown to reside in the synovium of patients with leukocyte-rich RA^16–18^, including a subpopulation of *CD34*^+^ sublining fibroblasts, which was decreased in LA. Another fibroblast population enriched in leukocyte-rich RA and characterized by higher expression of MHC Class II genes *IL6* and *CXCL12* appeared to also be enriched in LA^10^. However, this population was mainly characterized by IFN-stimulated and MHC Class I/II genes, and, therefore, this signature cannot definitively be attributed to fibroblasts (see Supplementary Fig. S10 online). Furthermore, comparison of SLE and RA synovial gene profiles indicated maintenance of a lining *CD55*^+^ fibroblast layer in LA and tissue repair/destruction mechanisms in RA. Therefore, LA may differ from RA and OA in which joint organ pathology is characterized by fibroblast-mediated tissue damage and, rather, be characterized by a loss of function or dysregulation of proinflammatory fibroblasts with destructive potential.

LA may also differentiate from RA in its immune cellularity and composition. A greater number of genes were found significantly altered in LA than in RA but a smaller portion of these transcripts could be attributed to immune/inflammatory cell populations, indicating an overall greater immune infiltrate in RA than in LA. Of the immune/inflammatory cell-specific transcripts identified, RA upregulated DEGs indicated increased T-cells, B-cells, NK/NKT-cells, and other lymphocytes, whereas LA upregulated DEGs were more characteristic of monocytes/macrophages and myeloid cells. Thus, LA may be more myeloid-mediated than RA. GSVA replicated this finding with significant upregulation of the core type I interferon signature, antigen presentation signature, inflammasome pathways, and monocyte/macrophage cell populations in LA including, notably, more inhibitors of inflammation. Specific macrophage and dendritic cell subsets originally identified in mouse synovium^12^ were also more enriched in LA. Interestingly, the downstream TNF, IL-1, and IL-6 signatures tended to be more enriched in LA than RA, indicating potential for repurposing anti-TNF biologics, the IL-1 antagonists anakinra and canakinumab, and IL-6R antagonist tocilizumab to treat LA.

Based on our data, TNF and IL-1 appear to have similar proinflammatory roles in driving LA. TNF and its receptors have been reported to be elevated in the serum of active SLE patients^19^, although treatment of SLE by TNF-blockers has been controversial up to this point. Anti-TNF therapies have, in some cases, induced lupus-like disease through skin manifestations and elevation of autoantibody levels and may exacerbate systemic disease through induction of apoptosis leading to increased exposure of nuclear antigens^20–23^. However, TNF neutralizing agents, including etanercept and infliximab, have been claimed to successfully treat LA^22–24^ and in a long-term observational study, only minor side effects and infrequent adverse events were observed^24^. Similarly, serum IL-1 levels have also been reported to correlate with disease activity^25^. TNF and IL-1 share several functions including promotion of local inflammation and induction of the expression of adhesion molecules and cytokines to attract proinflammatory leukocytes to sites of inflammation^26^. Two studies of small numbers of SLE patients indicated safety, tolerability, and overall efficacy of anakinra, one of which was focused on treating LA^27,28^. Controlled trials are necessary to inform further potential of inhibiting the TNF and IL-1 signaling axes in LA.

The detection of Ig heavy chain pre- and post-switch plasma cells in LA was notable. *IRF4*, *XBP1*, and *PRDM1*, genes essential for plasma cell maturation^29^, were all detected in LA. There was some evidence of GC formation in LA, but *BCL6*, *AICDA*, and *RGS13* were not upregulated nor detected in an LA-associated WGCNA module. However, *CXCL13*, encoding a chemoattractant that has been reported in RA synovial GCs^30^, was strongly upregulated. These findings suggest that fully-developed GCs are not a routine part of LA. Rather, it is more likely that lupus synovium contains lymphoid aggregates that support B-cell proliferation and autoantibody formation, as reported in the spleen in immune thrombocytopenia^31^. An interesting caveat to our data is the strong negative correlations of the midnightblue module, which contains the plasma cell signature, to SLEDAI and anti-dsDNA, whilst being positively correlated to LA. This suggests that the presence of plasmablasts/plasma cells in lupus synovium may not contribute significantly to systemic autoantibody levels and extra-articular lupus disease activity. Rather, the nature of the local inflammation may facilitate entry of circulating plasmablasts/plasma cells into the synovial space and/or their local differentiation.

The overexpression of numerous chemokines and chemokine receptors suggests chemokine signaling may play an important role in the infiltration of immune/inflammatory cells into lupus synovium. *CXCR3* and its ligands *CXCL9*, *CXCL10*, and *CXCL11* were all found upregulated and co-expressed in the midnightblue module, which contained a robust lymphocyte signature. This signaling axis is known to be induced by IFNγ and is involved in the recruitment of activated lymphocytes, particularly of naïve T-cells and their differentiation into T helper type I (Th1) cells^32^. *CXCR3* and *CXCR4*, both of which were upregulated in LA, are additionally important for the homing and maintenance of plasma cells^33^. These chemokine receptors could be involved in the recruitment of circulating plasmablasts/plasma cells into lupus synovium and/or their in situ retention and/or differentiation^34–36^. Other chemokines and their receptors such as *CCR5*–*CCL4/CCL5* could contribute to recruitment of other leukocytes into the synovium, including macrophages, monocytes, and T-cells^37^.

We utilized gene expression analysis to predict novel drugs that might target abnormally expressed genes or pathways and suppress inflammation. Predicted drugs and compounds identified novel potential therapies, but also confirmed current treatments by identifying standard-of-care lupus drugs such as glucocorticoids, methotrexate, aspirin, and cyclosporine. Notably, a large number of anti-cancer drugs with variable mechanisms of action were also predicted.

Drugs targeting the CDK family were predicted to revert the LA gene signature and may point to potential repurposing of drugs such as palbociclib or related seliciclib and other CDK inhibitors that have been shown to ameliorate nephritis in animal models^38^, possibly by reducing proliferation of lupus T- and B-cells in vitro^39^. Similarly, bucladesine was one of six phosphodiesterase inhibitors predicted to suppress LA. Other novel druggable therapeutic targets include GSK3, PARP1 and PARP2, and HDAC.

Notably, a large number of sodium channel blockers were predicted to target LA, possibly related to increased nervous innervation of the inflamed synovium. Neurologic targets included the acetylcholine, adrenergic, and glutamate receptors. These may have been predicted based on changes in the innervation of the inflamed tissue, although an effect on immune/inflammatory cells is also possible^40^.

There are several limitations to this study that need to be addressed. First, of importance, is the small size of the dataset. Although we have made statistical corrections for small sample size throughout our analyses as needed, these findings require validation in a larger cohort of LA patients. However, there are no other SLE synovium gene expression data that we know of and biopsy of lupus synovitis is rare. Second, given the nature of the bulk microarray data, identification of discrete cell types present is not certain and we have relied on specific transcriptomic signatures to identify relevant immune and inflammatory cell subtypes. As such, comparison to signatures identified by scRNA-seq must be interpreted with caution. Finally, of note, is that the majority of our analyses use OA as a noninflammatory arthritis comparator in the absence of healthy synovium samples.

Bioinformatic analysis of LA revealed a pattern of immunopathogenesis in which myeloid cell-mediated inflammation dominates. The breadth of the immune response underlying LA provides a basis for multiple avenues of therapeutic intervention to be considered that mouse models and previous studies have not provided. With these findings we can begin to hypothesize specific candidate target genes and pathways from which to develop or repurpose drugs to treat and improve LA specifically.

## Methods

### Gene expression data sourcing and patient characteristics

All data analyzed in this study were obtained from a publicly available gene set from synovial biopsies (NCBI Gene Expression Omnibus (GEO) GSE36700)^8^. No additional patient samples were employed. The SLE patients assessed had a mean (± s.d.) age of 32 years (9.49), SLEDAI of 8.25 (1.71), CRP of 12.5 mg/L (4.12), C3 of 82.5 mg/dL (28.0), C4 of 13 mg/dL (3.56) and anti-dsDNA of 97.6 IU/mL (77.0), and all patients had active arthritis at the time biopsy was taken. Complete patient data can be found in Supplementary Table S1 online. Data processing and analysis were conducted within the R statistical programming platform using relevant Bioconductor packages*.*

### Data normalization

All raw data files underwent background correction and GCRMA normalization resulting in log2 intensity values compiled into expression set objects (e-sets). Outliers were identified through the inspection of first, second, and third principal components and through inspection of array dendrograms calculated using Euclidean distances and clustered using average/UPGMA agglomeration. GSM899013_OA5 was consistently identified as an outlier and excluded from further analyses. Low intensity probes were removed by visual assignment of a 2.34 threshold cutoff upon a histogram of binned log2-transformed probe intensity values.

### Differential gene expression

Identification of DEGs in SLE vs OA samples (n = 8) and RA vs OA samples (n = 11) was conducted using the LIMMA package in R. To increase the probability of finding DEGs, both Affymetrix chip definition files (CDFs) and BrainArray CDFs were used to create and annotate e-sets, analyzed separately, then results merged. Linear models of normalized gene expression values were created through empirical Bayesian fitting. Resultant p-values were adjusted for multiple hypothesis testing using the Benjamini–Hochberg correction. Significant probes were filtered to retain a pre-specified False Discovery Rate (FDR) < 0.2 and duplicate probes were removed again to retain the most significant probe. The FDR was assigned a priori to avoid excluding false negative probes. The full list of DEGs can be found in Supplementary Data S1–2 online.

### Weighted gene co-expression network analysis (WGCNA)

The same normalized and filtered data (Affy CDFs only) were inputted into WGCNA to conduct an unsupervised clustering analysis yielding statistically co-expressed modules of genes used for further biological interrogation. Low-intensity probes were filtered as described to remove noise and help optimize the quality of the co-expression network. A scale-free topology matrix (TOM) was calculated to encode the network strength between probes with a soft thresholding power of 30. TOM distances were used to cluster probes into WGCNA modules. Resulting co-expression networks were trimmed using dynamic tree cutting and the deepSplit function in R. Partitioning around medoids (PAM) was also utilized to assign outliers to the nearest cluster. The resulting network was formed with a minimum module size of 100, cut height of 1, and merge height of 0.2. Modules were given random color assignments and expression profiles summarized by a module eigengene (ME). Final membership of probes representing the same gene were decided based on strongest within-module correlation to the ME value. For each module, ME values were correlated by Pearson correlation to clinical data including cohort, SLEDAI, anti-dsDNA, C3, C4, and CRP levels. Cohort was represented as a binary variable where SLE = 1 and OA = 0 whereas the remaining clinical data were continuous variables. Full module gene lists can be found in Supplementary Data S8–13 online.

### Quality control (QC) and selection of WGCNA modules

WGCNA modules of interest underwent a QC process to ensure modules were reflective of disease state. First, ME expression per patient was visually inspected to assess consistency of gene expression in a given cohort. Second, module membership, or eigengene-based connectivity (k_ME_), was plotted against probe correlation to the primary clinical trait of interest (SLEDAI) to gauge how well a given module agreed to the clinical trait. Finally, the Pearson correlations of MEs to the clinical metadata were examined. Absolute values of correlation coefficients in the range 0.5–1 were considered strong and alpha = 0.05 determined significance.

### Functional analysis

Immune/Inflammation-Scope (I-Scope) and Biologically Informed Gene Clustering (BIG-C) are functional aggregation tools for characterizing immune cells by type and biologically classifying large groupings of genes, respectively. I-Scope categorizes gene transcripts into a possible 32 hematopoietic cell categories based on matching 926 transcripts known to mark various types of immune/inflammatory cells. BIG-C sorts genes into 52 different groups based on their most probable biological function and/or cellular/subcellular localization. Tissue-Scope (T-Scope) is an additional aggregation tool to characterize cell types found in specific tissues. In these analyses only the two T-Scope categories relevant to the synovium were used: fibroblasts and synoviocytes. I-Scope and T-Scope were utilized to calculate enrichment of cellular signatures and BIG-C was utilized to calculate enrichment of functional signatures of the LA gene expression profile.

### Network analysis

Cytoscape (V3.6.1) software was used to visualize protein–protein interactions based on the Search Tool for the Retrieval of Interacting Genes/Proteins (STRING) database via the stringApp plugin application. A confidence score of 0.40 was used. The clustermaker2 plugin application was used to created MCODE clusters of interrelated genes using a network scoring degree cutoff of 2, node score cutoff of 0.2, maximum depth of 100, and k-Core of 2. Genes not recognized by the STRING database were removed from datasets prior to upload into Cytoscape.

### Ingenuity pathway analysis (IPA)

The canonical pathway and UPR functions of IPA core expression analysis (Qiagen) were used to interrogate DEGs and WGCNA module gene lists. Core expression analyses were based on fold change if uploaded genes were differentially expressed; otherwise, a fold change of one was used. Canonical pathways and UPRs were considered significant if |Activation Z-Score| ≥ 2 and overlap p-value < 0.01.

### Gene set variation analysis (GSVA)

The GSVA R package was used as a non-parametric, unsupervised gene set enrichment method. Enrichment scores were calculated using a Kolgomorov Smirnoff (KS)-like random walk statistic to estimate variation of pre-defined gene sets. The inputs for the GSVA algorithm were e-sets containing log2 microarray expression values (Affy HGU133plus2 definitions) and pre-defined gene sets co-expressed in SLE datasets. Low-intensity probes were filtered out based on interquartile range (IQR)^41^. GSVA was conducted on the remaining network and Welch’s *t* test was used to detect significant differences in enrichment between cohorts at an alpha level of 0.05, followed by calculation of Hedge’s g effect size with correction for small samples. Welch’s *t* test was used to account for unequal variances in both the SLE and OA populations and RA and OA populations.

Enrichment gene sets containing cell type- and process-specific genes listed in Supplementary Data S14 online were created through an iterative process of identifying DE transcripts pertaining to a restricted profile of hematopoietic cells in 13 SLE microarray datasets and checked for expression in purified T-cells, B-cells, and monocytes to remove transcripts indicative of multiple cell types. Genes were identified through literature mining, gene ontology (GO) biological pathways, and STRING interactome analysis as belonging to specific categories^42^. Select gene sets were derived directly from in vitro experiments^43,44^. The M1 signature was edited to remove interferon stimulated genes. Additionally, IL-1 and IL-6 gene sets were derived from the first three tiers of the respective PathCards signaling pathways.

### Co-expression analysis

Co-expression analyses of literature-derived signatures published in mouse and human synovium were conducted in R. Briefly, Spearman’s rank correlation coefficients and p-values were computed using the rcorr() function based upon input log2 expression values for each gene in each SLE and OA sample. Spearman correlations were chosen to avoid assuming linear relationships. The input gene signature was refined to contain genes significantly correlating with at least 25% of the original gene signature at an alpha level of 0.05, then refined again to contain genes positively correlated with at least 25% of the new signature (i.e., Spearman’s rho > 0). The final co-expressed signatures were used as GSVA gene sets. Mouse to human ortholog conversion was done using the homologene R package.

### LINCS drug–target prediction and biological upstream regulator analysis

The LINCS perturbation database (https://data.lincscloud.org.s3.amazonaws.com/index.html) is a database of transcriptional signatures generated from functional perturbations (i.e., gene overexpression, gene knockdown, or treatment with drugs/compounds) in over 25 reference cell lines to which a user can upload and compare a gene expression signature of interest and determine connectivity scores to specific perturbations. We queried this connectivity mapping database using a list of significantly up- and down-regulated genes from the SLE and OA samples. Comparisons were made based on the LINCS-computed connectivity scores, where − 100 describes a transcriptional program perfectly opposing the user-uploaded gene signature and 100 describes a transcriptional program perfectly representative of the user-uploaded gene signature. BURs were identified by the knocked-down and overexpressed gene transcripts that resulted in connectivity scores in the − 75 to − 100 and 50 to 100 ranges, respectively. Compounds resulting in connectivity scores in the − 75 to − 100 ranges were analyzed and summarized by drug target.

### Drug–target matching

LINCS-predicted BURs and IPA-predicted UPRs were annotated with respective targeting drugs and compounds to elucidate potential useful therapies in lupus synovitis (see Supplementary Data S15–16 online). Drugs targeting gene products of interest both directly and indirectly were sourced by IPA, the Connectivity Map via the drug repurposing tool, GeneCards, Search Tool for Interactions of Chemicals (STITCH) database (V5.0), Combined Lupus Treatment Scoring (CoLTS)-scored drugs^45^, FDA labels, DrugBank, literature mining, and queries of clinical trials databases. The drug repurposing tool was accessed at https://clue.io/repurposing-app.

### STITCH

The (STITCH) (V5.0) database (https://stitch.embl.de/) of known and predicted protein–protein and protein–chemical interactions was used to predict direct and indirect drug targeting mechanisms. For each gene product of interest, the top 10 interactors were analyzed and drugs directly targeting the top interactors were matched according to the methods described. A medium confidence score cutoff of 0.4 for interaction predictions was used. Predicted interactions based solely on text-mining were not considered.

### Statistical analysis

Enrichment statistics in SLE vs OA were calculated by right-sided Fisher’s Exact Test in R using the function fisher.test(). Statistical significance was obtained at p < 0.05.

## Supplementary information

Supplementary information 1.

Supplementary information 2.

## Data Availability

The dataset analyzed during the current study is available in the NCBI GEO repository, https://www.ncbi.nlm.nih.gov/geo/query/acc.cgi?acc=GSE36700. Additional data generated from analyses are included in this published article (and its Supplementary Information files).

## References

[1] Dörner T, Jacobi AM, Lipsky PE (2009). B cells in autoimmunity. Arthritis Res. Ther..

[2] Grossman J (2009). Lupus arthritis. Best Pract. Res. Clin. Rheumatol..

[3] Ball E, Gibson D, Bell A, Rooney M (2013). Plasma IL-6 levels correlate with clinical and ultrasound measures of arthritis in patients with systemic lupus erythematosus. Lupus.

[4] Eilertsen G, Nikolaisen C, Becker-Merok A, Nossent J (2011). Interleukin-6 promotes arthritis and joint deformation in patients with systemic lupus erythematosus. Lupus.

[5] Illei GG (2010). Tocilizumab in systemic lupus erythematosus: data on safety, preliminary efficacy, and impact on circulating plasma cells from an open-label phase I dosage-escalation study. Arthritis Rheumatol..

[6] Hoffman IEA (2004). Specific antinuclear antibodies are associated with clinical features in systemic lupus erythematosus. Ann. Rheum. Dis..

[7] Cozzani E, Drosera M, Gasparini G, Parodi A (2014). Serology of lupus erythematosus: correlation between immunopathological features and clinical aspects. Autoimmune Dis..

[8] Lauwerys, B. R. Gene expression profiles in synovial biopsies from patients with arthritis. Dataset. *NCBI GEO.* GSE36700 https://www.ncbi.nlm.nih.gov/geo/query/acc.cgi?acc=GSE36700 (2012).

[9] Nzeusseu-Toukap A (2007). Identification of distinct gene expression profiles in the synovium of patients with systemic lupus erythematosus. Arthritis Rheumatol..

[10] Zhang F (2019). Defining inflammatory cell states in rheumatoid arthritis joint synovial tissues by integrating single-cell transcriptomics and mass cytometry. Nat. Immunol..

[11] Kuo D (2019). HBEGF+ macrophages in rheumatoid arthritis induce fibroblast invasiveness. Sci. Transl. Med..

[12] Culemann S (2019). Locally renewing resident synovial macrophages provide a protective barrier for the joint. Nature.

[13] Christensen AD, Haase C, Cook AD, Hamilton JA (2016). K/BxN serum-transfer arthritis as a model for human inflammatory arthritis. Front. Immunol..

[14] Labonte AC (2018). Identification of alterations in macrophage activation associated with disease activity in systemic lupus erythematosus. PLoS ONE.

[15] Lauwerys BR (2015). Heterogeneity of synovial molecular patterns in patients with arthritis. PLoS ONE.

[16] Kim EK (2017). IL-17-mediated mitochondrial dysfunction impairs apoptosis in rheumatoid arthritis synovial fibroblasts through activation of autophagy. Cell Death Dis..

[17] Wang S, Wang L, Wu C, Sun S, Pan JH (2018). E2F2 directly regulates the STAT1 and PI3K/AKT/NF-κB pathways to exacerbate the inflammatory phenotype in rheumatoid arthritis synovial fibroblasts and mouse embryonic fibroblasts. Arthritis Res. Ther..

[18] Mizoguchi F (2018). Functionally distinct disease-associated fibroblast subsets in rheumatoid arthritis. Nat. Commun..

[19] Studnicka-Benke A, Steiner G, Petera P, Smolen JS (1996). Tumour necrosis factor alpha and its soluble receptors parallel clinical disease and autoimmune activity in systemic lupus erythematosus. Br. J. Rheumatol..

[20] Blasco-Morente G, Notario-Ferreira I, Rueda-Villafranca B, Tercedor-Sánchez J (2015). Subacute cutaneous lupus erythematosus induced by golimumab. Med. Clín. (English Ed.).

[21] Wilkerson E, Hazey MA, Bahrami S, Callen JP (2012). Golimumab-exacerbated subacute cutaneous lupus erythematosus. Arch. Dermatol..

[22] Yang X, Zhu LJ, Yu XQ (2010). Anti-TNF-α therapies in systemic lupus erythematosus. J. Biomed. Biotechnol..

[23] Swale VJ, Perrett CM, Denton CP, Black CM, Rustin MHA (2003). Etanercept-induced systemic lupus erythematosus. Clin. Exp. Dermatol..

[24] Cortés-Hernández J, Egri N, Vilardell-Tarrés M, Ordi-Ros J (2015). Etanercept in refractory lupus arthritis: an observational study. Semin. Arthritis Rheum..

[25] Brugos B (2010). Measurement of interleukin-1 receptor antagonist in patients with systemic lupus erythematosus could predict renal manifestation of the disease. Hum. Immunol..

[26] Davis LS, Hutcheson J, Mohan C (2011). The role of cytokines in the pathogenesis and treatment of systemic lupus erythematosus. J. Interf. Cytokine Res..

[27] Ostendorf B (2005). Preliminary results of safety and efficacy of the interleukin 1 receptor antagonist anakinra in patients with severe lupus arthritis. Ann. Rheum. Dis..

[28] Moosig F, Zeuner R, Renk C, Schröder JO (2004). IL-1RA in refractory systemic lupus erythematosus. Lupus.

[29] Tellier J (2016). Blimp-1 controls plasma cell function through the regulation of immunoglobulin secretion and the unfolded protein response. Nat. Immunol..

[30] Shi K (2001). Lymphoid chemokine B cell-attracting chemokine-1 (CXCL13) is expressed in germinal center of ectopic lymphoid follicles within the synovium of chronic arthritis patients. J. Immunol..

[31] Daridon C (2012). Splenic proliferative lymphoid nodules distinct from germinal centers are sites of autoantigen stimulation in immune thrombocytopenia. Blood.

[32] Tokunaga R (2018). CXCL9, CXCL10, CXCL11/CXCR3 axis for immune activation—a target for novel cancer therapy. Cancer Treat. Rev..

[33] Muehlinghaus G (2005). Regulation of CXCR3 and CXCR4 expression during terminal differentiation of memory B cells into plasma cells. Blood.

[34] Buckley CD (2000). Persistent induction of the chemokine receptor CXCR4 by TGF-β1 on synovial T cells contributes to their accumulation within the rheumatoid synovium. J. Immunol..

[35] Tsubaki T (2005). Accumulation of plasma cells expressing CXCR3 in the synovial sublining regions of early rheumatoid arthritis in association with production of Mig/CXCL9 by synovial fibroblasts. Clin. Exp. Immunol..

[36] Henneken M, Dörner T, Burmester GR, Berek C (2005). Differential expression of chemokine receptors on peripheral blood B cells from patients with rheumatoid arthritis and systemic lupus erythematosus. Arthritis Res. Ther..

[37] de Oliveira CEC (2014). CC chemokine receptor 5: the interface of host immunity and cancer. Dis. Markers.

[38] Zoja C (2007). Cyclin-dependent kinase inhibition limits glomerulonephritis and extends lifespan of mice with systemic lupus. Arthritis Rheumatol..

[39] Goulvestre C (2005). A mimic of p21WAF1/CIP1 ameliorates murine lupus. J. Immunol..

[40] Dantzer R (2018). Neuroimmune interactions: from the brain to the immune system and vice versa. Physiol. Rev..

[41] Chockalingam, S., Aluru, M. & Aluru, S. Microarray data processing techniques for genome-scale network inference from large public repositories. *Microarrays *(*Basel, Switzerland*)**5** (2016). 10.3390/microarrays5030023 PMC5040970 27657141

[42] Catalina MD, Bachali P, Geraci NS, Grammer AC, Lipsky PE (2019). Gene expression analysis delineates the potential roles of multiple interferons in systemic lupus erythematosus. Commun. Biol..

[43] Waddell SJ (2010). Dissecting interferon-induced transcriptional programs in human peripheral blood cells. PLoS ONE.

[44] Martinez FO, Gordon S, Locati M, Mantovani A (2006). Transcriptional profiling of the human monocyte-to-macrophage differentiation and polarization: new molecules and patterns of gene expression. J. Immunol..

[45] Grammer AC (2016). Drug repositioning in SLE: crowd-sourcing, literature-mining and big data analysis. Lupus.

[46] Hubbard, E. *et al.* Analysis of lupus synovitis gene expression reveals dysregulation of pathogenic pathways activated within infiltrating immune cells—ACR Meeting Abstracts (2018). https://acrabstracts.org/abstract/analysis-of-lupus-synovitis-gene-expression-reveals-dysregulation-of-pathogenic-pathways-activated-within-infiltrating-immune-cells/. (Accessed: 17th December 2019)

[47] Hubbard, E. *et al.* Analysis of gene expression from systemic lupus erythematosus synovium reveals unique pathogenic mechanisms—ACR Meeting Abstracts (2019). https://acrabstracts.org/abstract/analysis-of-gene-expression-from-systemic-lupus-erythematosus-synovium-reveals-unique-pathogenic-mechanisms/. (Accessed: 17th December 2019)

