# Supplementary material for: Analysis of gene expression from systemic lupus erythematosus synovium reveals myeloid cell-driven pathogenesis of lupus arthritis
Source: Sci Rep. 2020 Oct 15;10:17361. doi: 10.1038/s41598-020-74391-4 (PMC7562741; doi:10.1038/s41598-020-74391-4)
Supplement: Supplementary file 2 — Supplementary information 2. [file 41598_2020_74391_MOESM2_ESM.pdf]

Supplementary Information

Analysis of Gene Expression from Systemic Lupus Erythematosus Synovium Reveals Myeloid Cell-Driven Pathogenesis of Lupus Arthritis

Erika L. Hubbard, Michelle D. Catalina, Sarah Heuer, Prathyusha Bachali, Robert Robl, Nicholas S. Geraci, Amrie C. Grammer, Peter E. Lipsky

Supplementary Table S1. Patient Characteristics

Accession	Title	Cohort	Age	Sex	SLEDAI	CRP	C3	C4	anti.dsDNA	ANA	ENA	Rheumatoid Factor	ACPA	Treatment
GSM898470	OA1	OA	71	F										NSAIDs
GSM899010	OA2	OA	73	F										
GSM899011	OA3	OA	65	F										NSAIDs
GSM899012	OA4	OA	51	F										NSAIDs
GSM899013	OA5	OA	56	M										
GSM899014	RA1	RA	52	M										NSAIDs
GSM899015	RA2	RA	42	M										
GSM899016	RA3	RA	43	F										
GSM899017	RA4	RA	69	M										NSAIDs
GSM899018	RA5	RA	55	M										
GSM899019	RA6	RA	62	M										NSAIDs
GSM899020	RA7	RA	37	F										NSAIDs
GSM899021	SLE1	SLE	38	F	10	12	79	16	193	1:640	Neg	Neg	Neg	
GSM899022	SLE2	SLE	19	F	6	8	123	15	5.6	1:60	Neg	Neg		NSAIDs
GSM899023	SLE3	SLE	40	F	8	18	62	8	85.7	1:20	Anti-RNP	Pos	Neg	NSAIDs
GSM899024	SLE4	SLE	31	F	9	12	66	13	106	1:60	Anti-RNP, anti-Ro SSA	Pos	Neg	

Clinical metadata of individual patients from whom synovial biopsies were collected. All patients had active articular disease at the time of biopsy. Units are as follows: Age (years), CRP (mg/L), C3 (mg/dL), C4 (mg/dL), and anti-dsDNA (IU/mL).

Supplementary Figure S1. WGCNA Reveals Six LA-Associated Modules of Genes

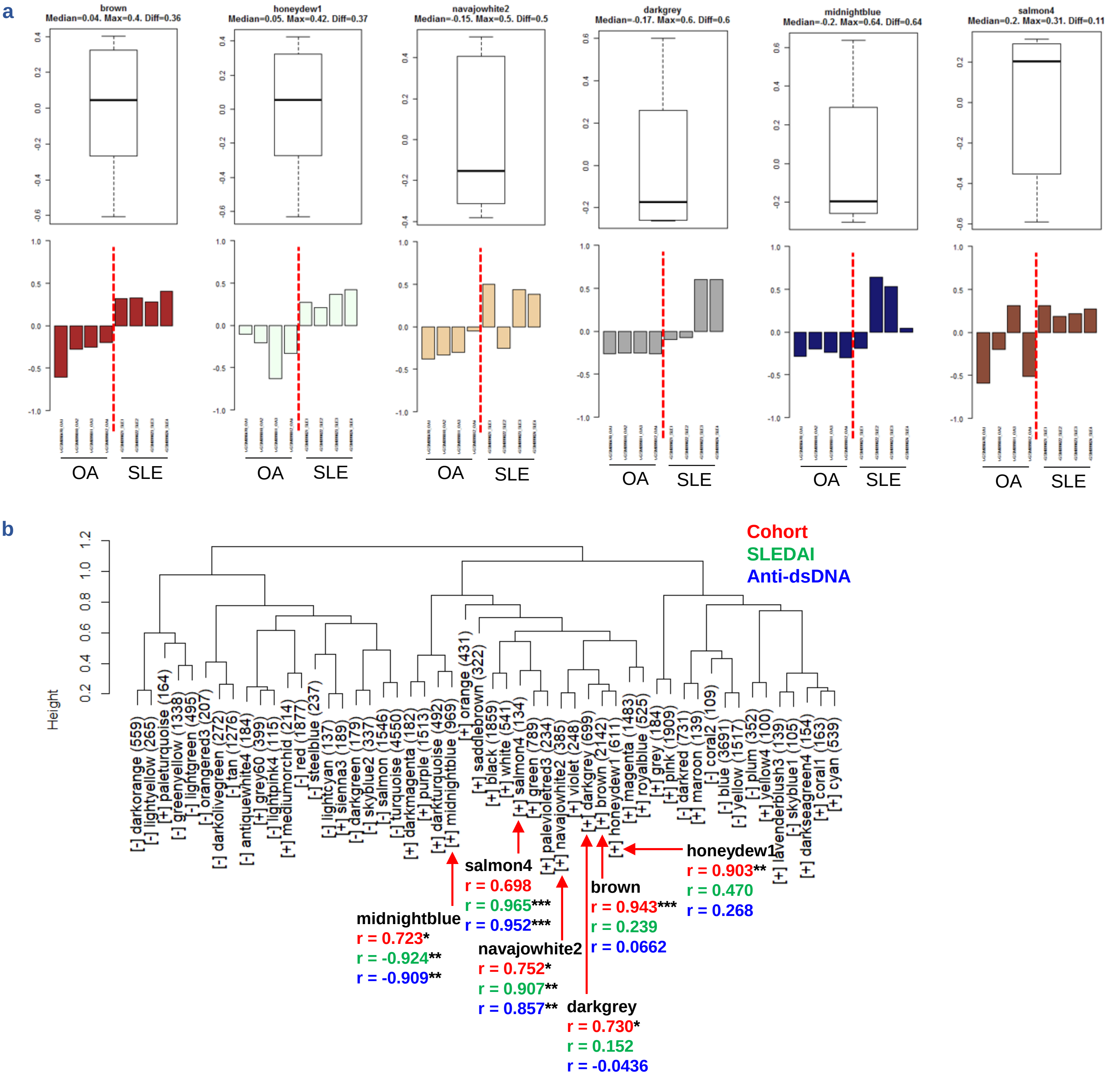

WGCNA of SLE vs OA patients yielded six modules of genes associated with LA after quality control. **(a)** Module eigengene plots per sample of the six LA-associated modules; color names are randomly generated as part of WGCNA module assignment. **(b)** Dendrogram of all 52 modules of co-expressed genes from WGCNA. Modules are labeled with the correlation direction to LA in brackets, the randomly generated color name of the module, and the number of microarray probes contained in the module in parentheses ([correlation] module name (# probes)). The six modules associated with LA are indicated with red arrows. Pearson correlation values to cohort, SLEDAI, and anti-dsDNA titer are shown in red, green, and blue, respectively. Significant correlations are marked: *p<0.05, **p<0.01, ***p<0.001.

Supplementary Figure S2. Quality Control of WGCNA Results

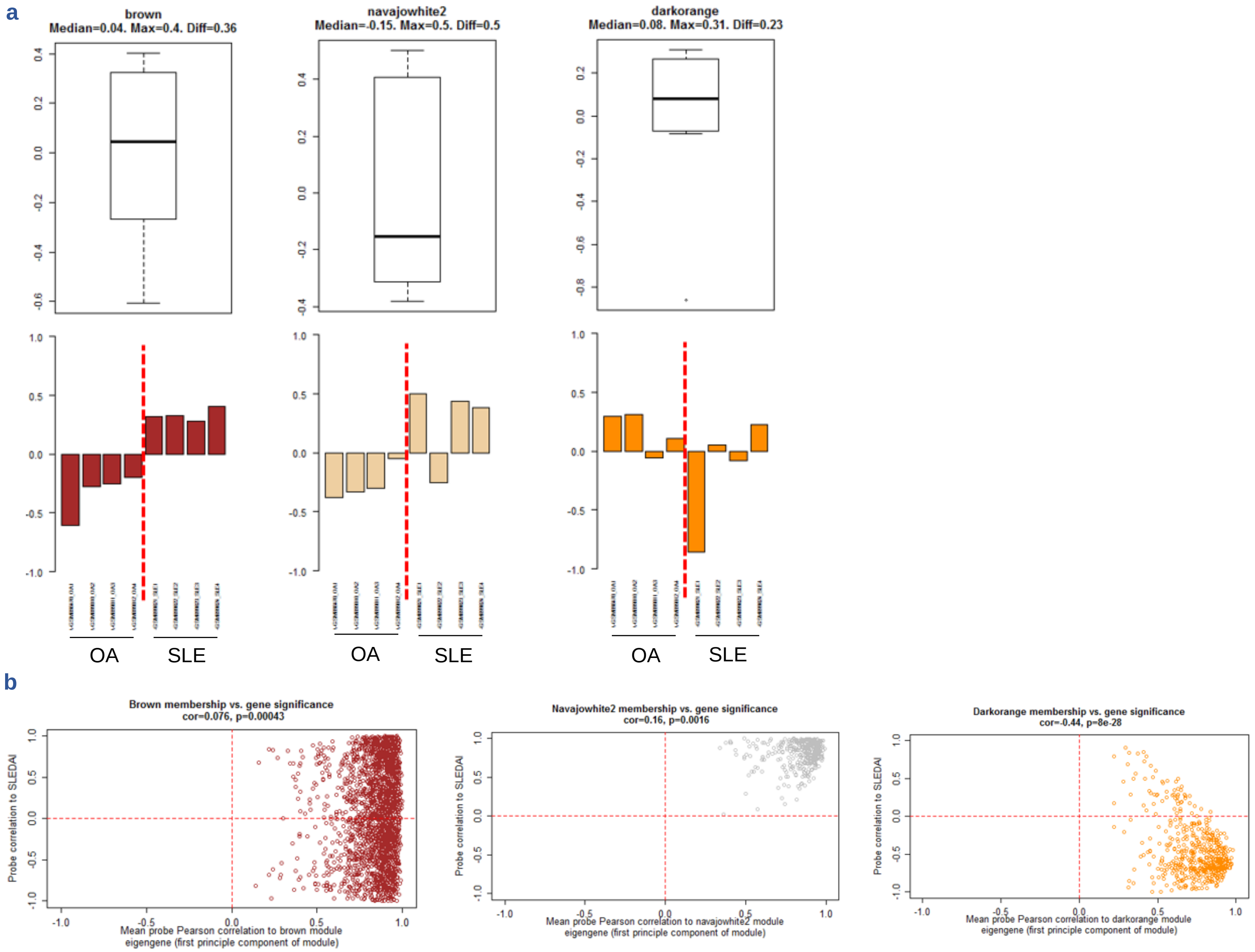

Two of the three WGCNA outputs contributing to quality control for the selection of modules for further interrogation are summarized here with three sample modules: brown, navajowhite2, and darkorange. **(a)** Module eigengene bar plots per patient sample in a module show variance of gene expression within a cohort. **(b)** Module membership (kME) plotted against individual probe correlation to SLEDAI shows agreement of individual module probes to trait correlation.

Supplementary Table S2. Sample WGCNA Module Eigengene Correlations

Module	cohort		SLEDAI		Anti-dsDNA		C3		C4		CRP	
	r	p	r	p	r	p	r	p	r	p	r	p
brown	0.943	4.3e-4	0.239	0.569	0.0662	0.876	-0.0914	0.830	0.375	0.360	-0.437	0.279
navajowhite2	0.752	0.0315	0.907	0.00186	0.857	0.00653	-0.928	8.90e-4	-0.314	0.448	0.714	0.0468
darkorange	-0.468	0.243	-0.582	0.130	-0.758	0.0294	0.0771	0.856	-0.401	0.325	-0.0644	0.880

Pearson correlations to clinical metadata, the final of three WGCNA outputs contributing to quality control for the selection of modules for further interrogation, are summarized here with three sample modules: brown, navajowhite2, and darkorange. Pearson correlation coefficients with corresponding p-values are described. Red and blue text indicate r values of significant correlations and anticorrelations ($p<0.05$), respectively.

Supplementary Figure S3. Immune Cell Enrichment in LA-Associated WGCNA Modules

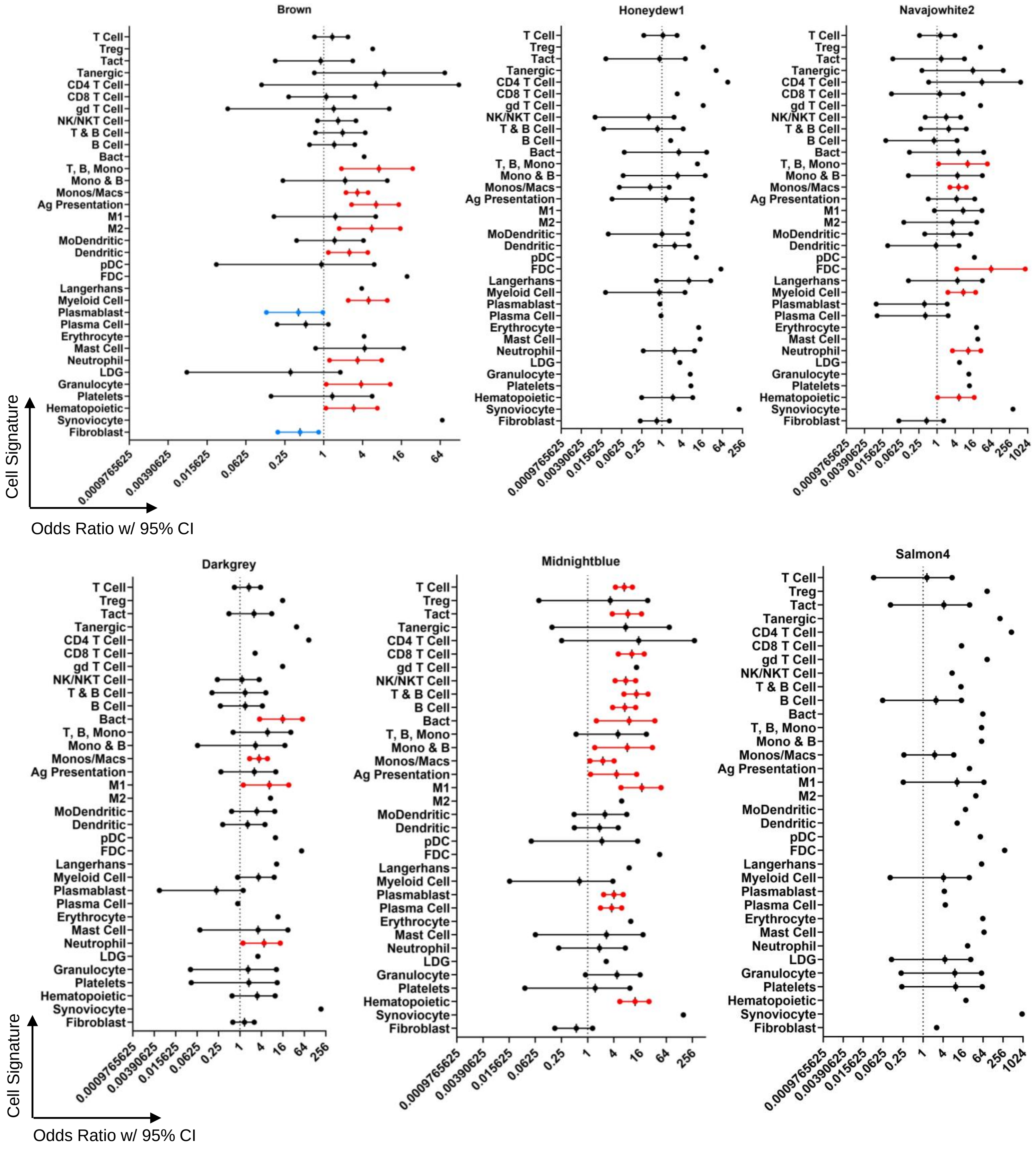

Odds ratios bound by 95% confidence intervals show specific immune/inflammatory cell populations or synovium-specific cell populations that could be linked to lupus synovitis. Significant enrichment ($p<0.05$) and confidence intervals that exclude odds ratio = 1 are colored red and blue for positive and negative association with the sample, respectively. X-axes are plotted on a log2 scale. For categories represented by a single point, odds ratio = 0 and the data point shown represents the upper bound of the confidence interval.

Supplementary Figure S4. Characterization of WGCNA Modules Negatively Correlated with LA

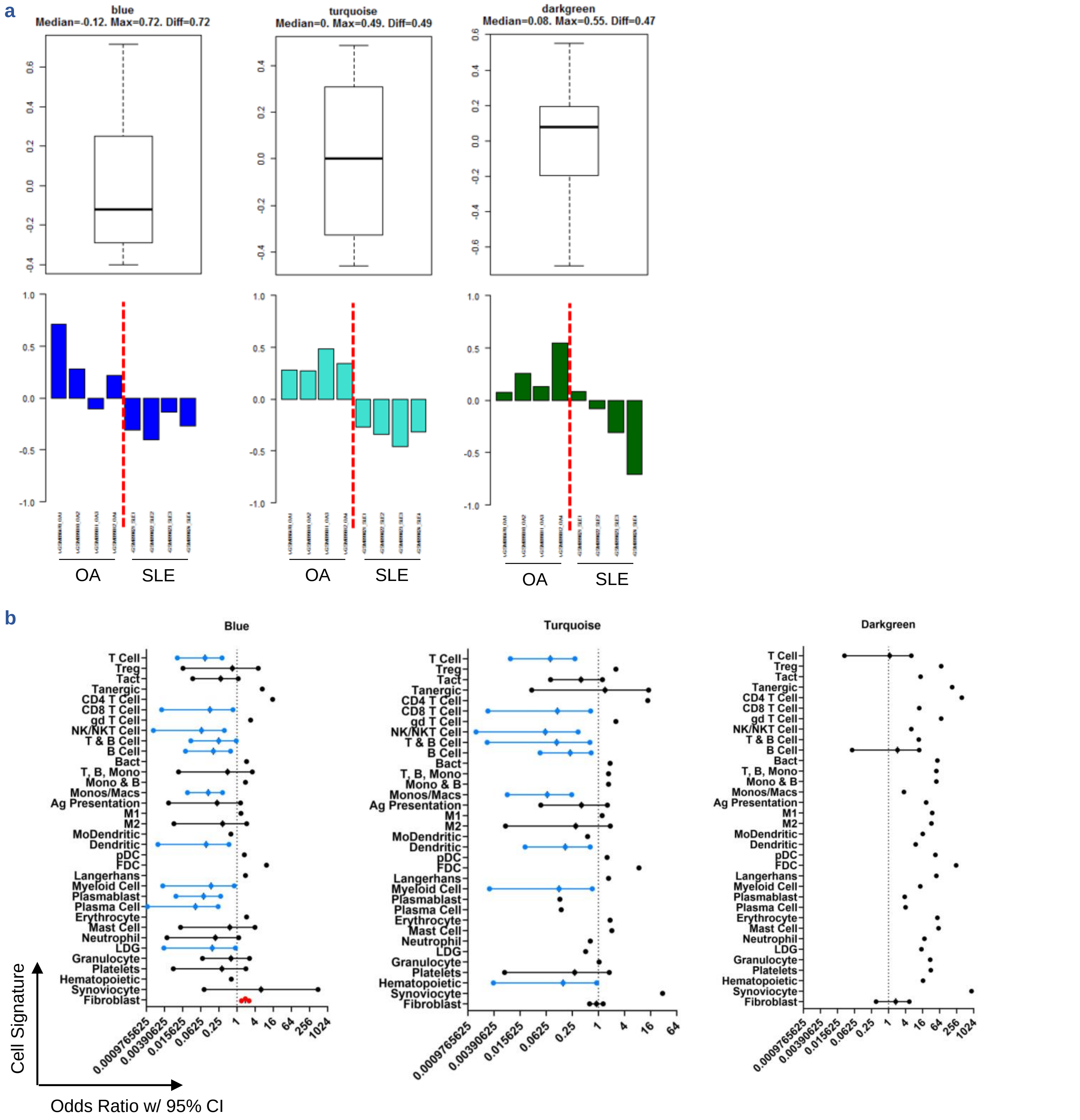

Overview of three WGCNA modules (blue, turquoise, darkgreen) negatively correlated with SLE. **(a)** Module eigengene bar plots per patient per cohort. **(b)** Enrichment of specific immune/inflammatory and synovial tissue cell populations. Significant enrichment ($p<0.05$) and a 95% confidence interval that excludes odds ratio = 1 are colored red and blue for positive and negative association with the sample, respectively. The x-axes are plotted on log2 scales. For categories represented by a single point, odds ratio = 0 and the data point shown represents the upper bound of the confidence interval.

Supplementary Figure S5. Immunoglobulin Transcripts Identified in LA

a

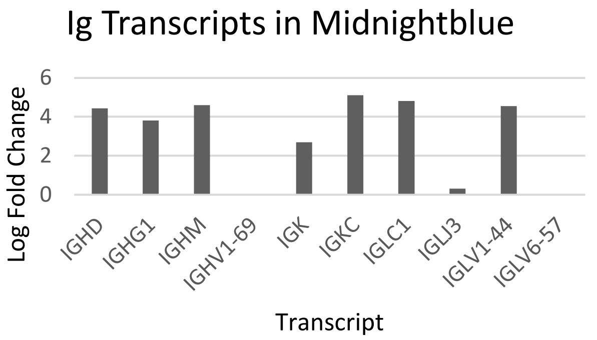

b

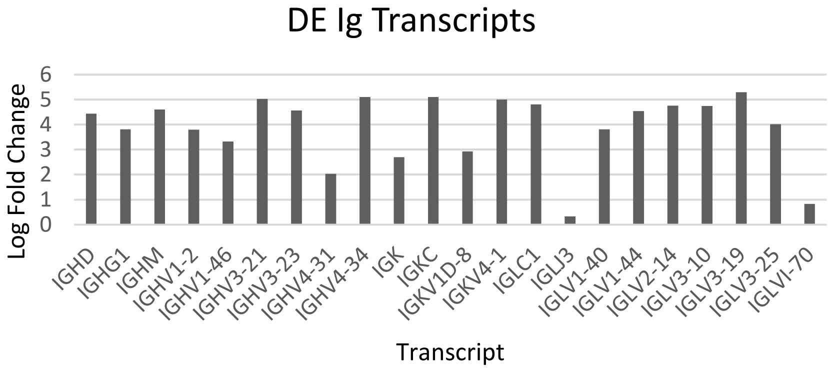

Immunoglobulin (Ig) transcripts detected in SLE vs OA synovium. **(a)** Log fold changes of Ig transcripts found in the LA-correlated WGCNA module containing the plasmablast/plasma cell signature, midnightblue. **(b)** Log fold changes of all Ig transcripts identified by differential expression analysis.

Supplementary Table S3. WGCNA Modules Negatively Correlated with LA

Module	cohort		SLEDAI		Anti-dsDNA		C3		C4		CRP	
	r	p	r	p	r	p	r	p	r	p	r	p
blue	-0.791	0.0195	0.344	0.404	0.261	0.533	-0.865	0.00557	-0.894	0.00274	0.985	1.00e-5
turquoise	-0.975	4.00e-5	0.395	0.333	0.430	0.288	0.284	0.495	0.942	4.70e-4	-0.717	0.0454
darkgreen	-0.716	0.0460	-0.0778	0.855	0.159	0.707	0.509	0.198	0.449	0.264	-0.247	0.555

Pearson correlation coefficients with corresponding p-values of all WGCNA modules significantly anticorrelated with LA. Red and blue text indicate r values of significant ($p<0.05$) correlations and negative correlations, respectively.

Supplementary Figure S6. Functional Enrichment in LA-Associated WGCNA Modules

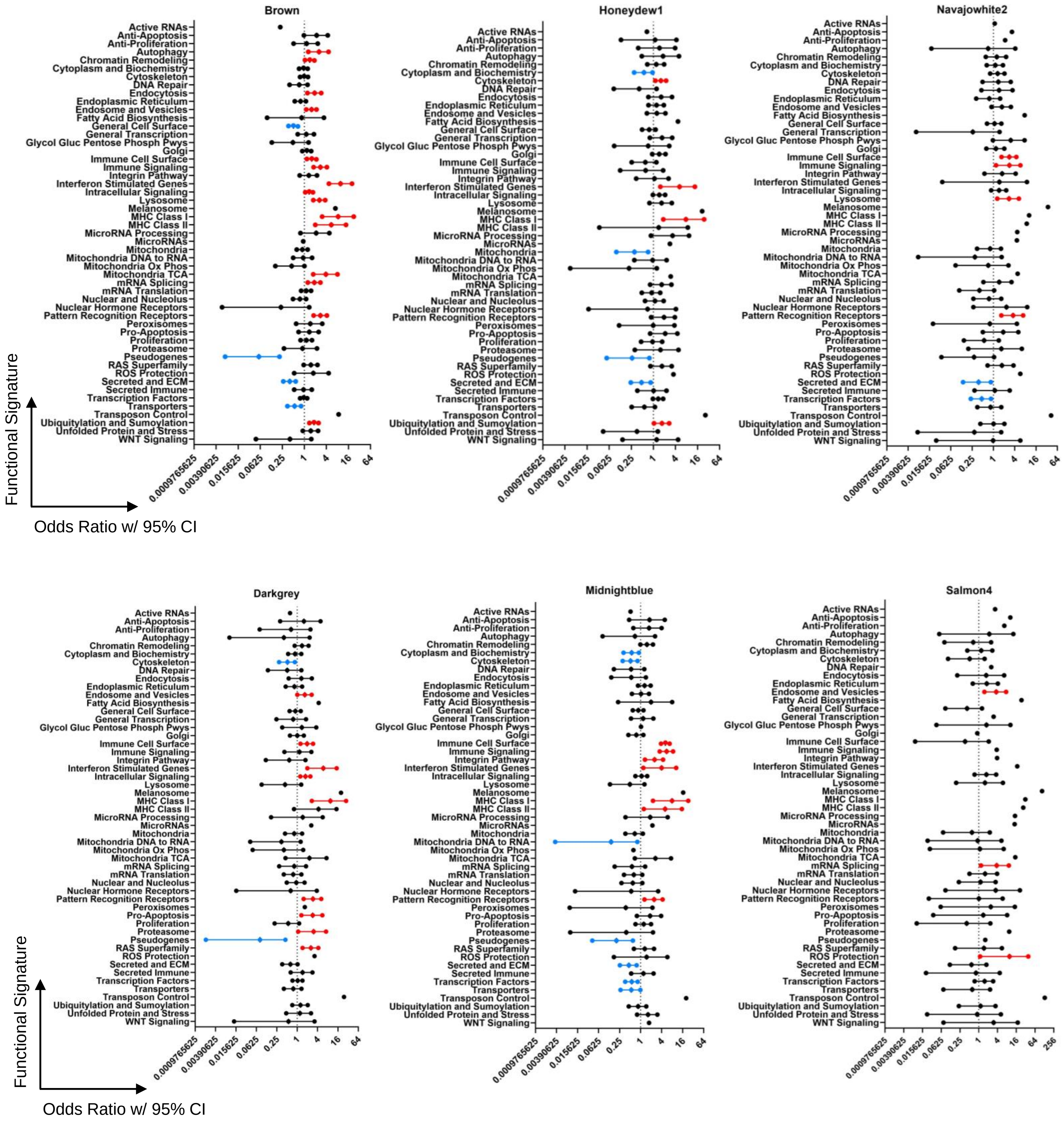

Odds ratios bound by 95% confidence intervals indicate enrichment of functional gene categories in six LA-associated WGCNA modules. Significant enrichment ($p<0.05$) and confidence intervals that exclude odds ratio = 1 are colored red and blue for positive and negative association with the sample, respectively. X-axes are plotted on a log2 scale. For categories represented by a single point, odds ratio = 0 and the data point shown represents the upper bound of the confidence interval.

Supplementary Figure S7. Functional Characterization of LA-Associated WGCNA Modules

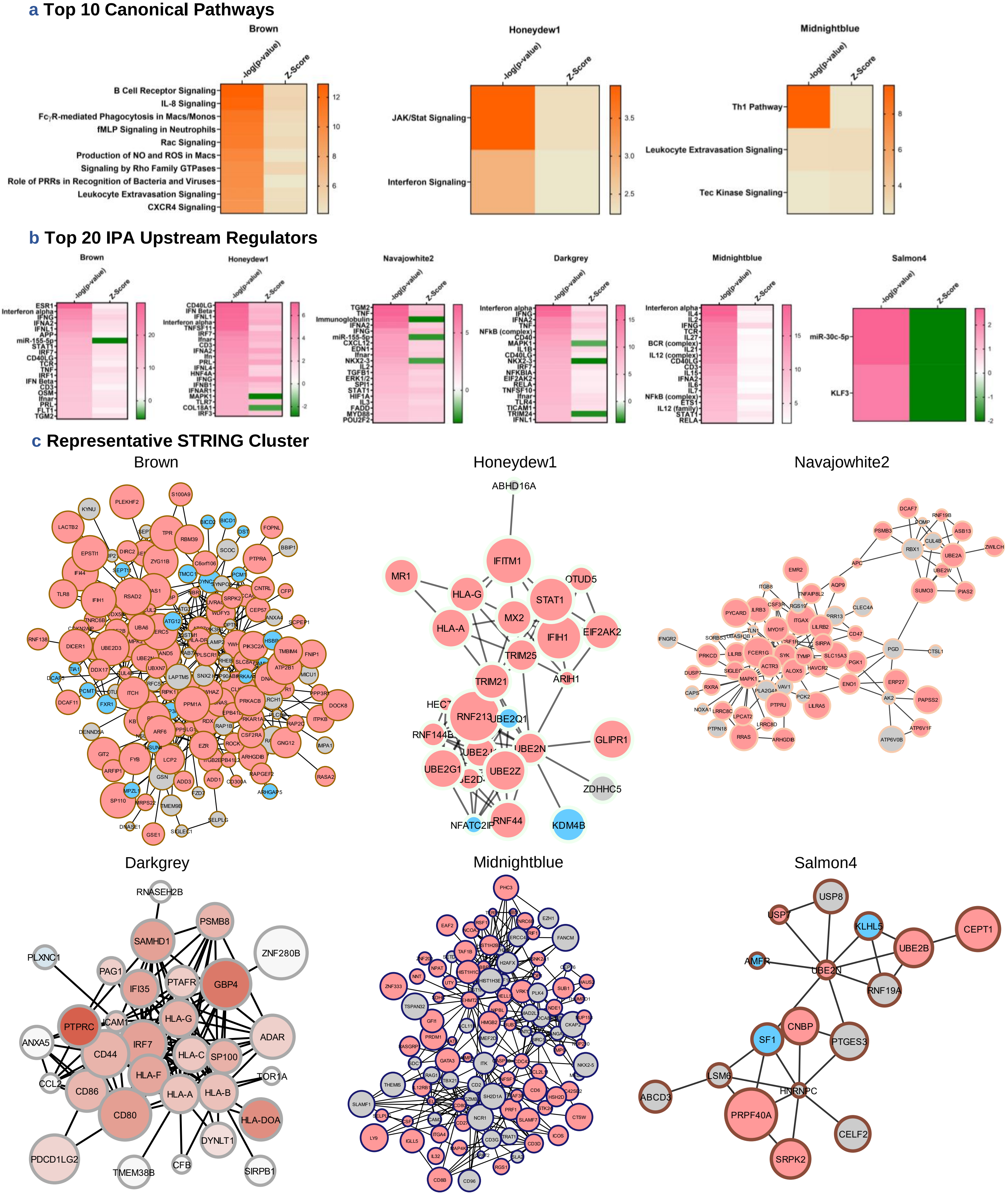

(a) Top 10 IPA canonical pathways for each of the six LA-associated WGCNA modules. Modules not shown were not predicted to have any significant pathways by p-value ($p<0.01$) and Activation Z-Score ($|Z\text{-Score}| \geq 2$) with biological relevance. **(b)** Top 20 IPA upstream regulators for each LA-associated WGCNA module. All IPA predictions in **(a, b)** are ordered by significance ($p<0.01$) and must have an $|\text{Activation Z-Score}| \geq 2$. **(c)** Single representative clusters from STRING depicting the protein-protein interactome of each LA-associated module. Clusters were chosen based on the highest number of intra-cluster connections and/or biological significance.

Supplementary Figure S8. Germinal Center B Cell and T_{fh} Cell Markers in LA

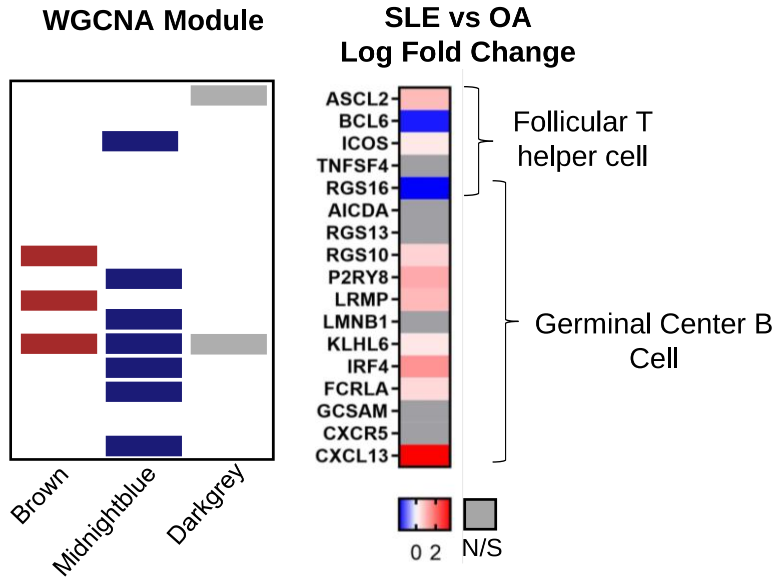

Assessment of GC B cell and T_{fh} cell markers in lupus synovium from differential expression analysis and WGCNA. Genes found in SLE-associated WGCNA modules are indicated. LIMMA log fold changes indicate markers that are DEGs.

Supplementary Figure S9. Comparison of Tissue-Specific Cell Types and Processes in SLE and RA Synovitis

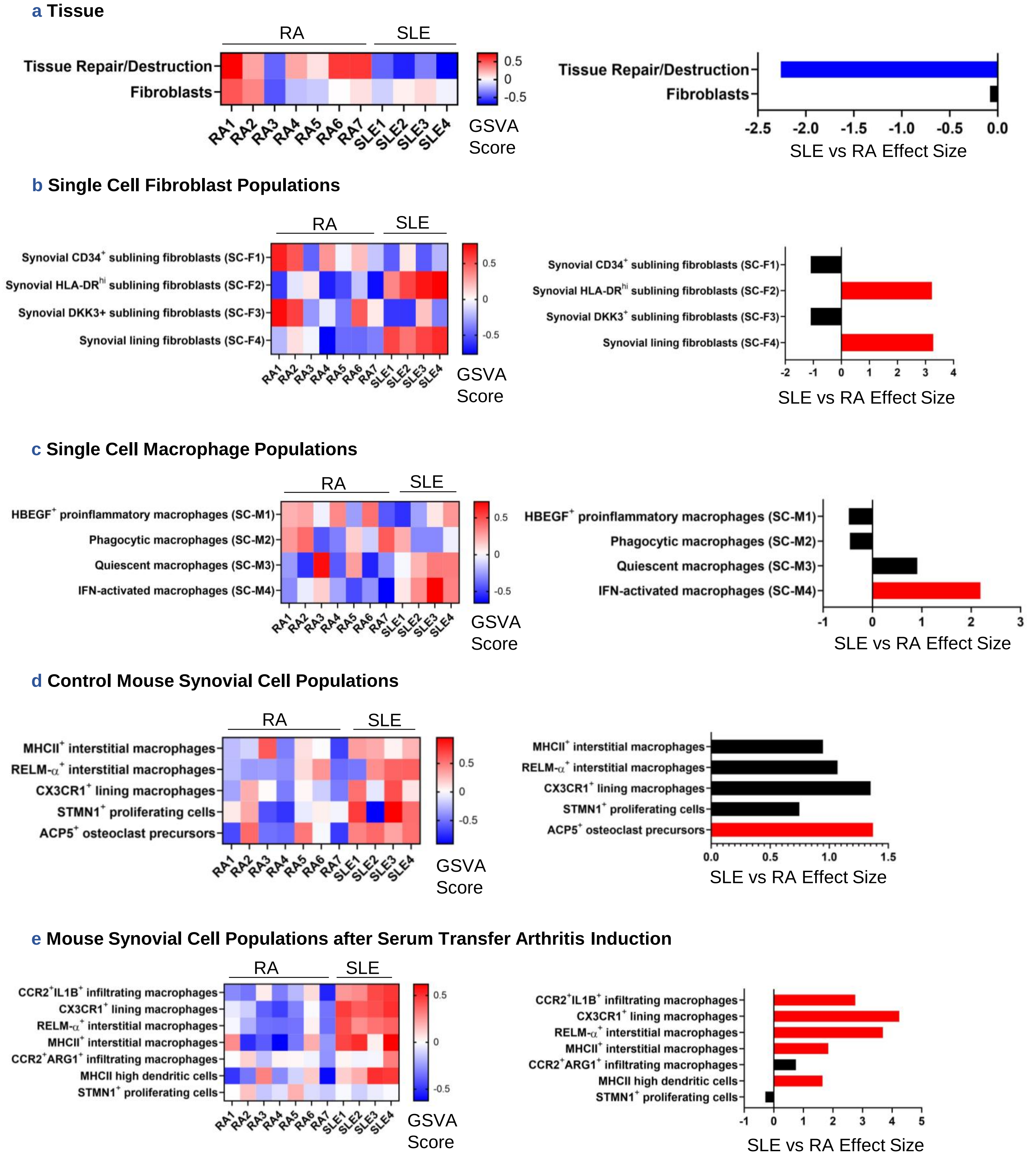

GSVA of synovial tissue processes and specific cell types **(a)** and literature-derived synovium-specific cell subtypes in human RA, OA, and mouse synovium **(b-e)** was conducted on log2-normalized gene expression values from SLE and RA synovium. Hedge's g effect sizes were calculated with correction for small sample size for each gene set and significant differences in enrichment between cohorts were found by Welch's t-test ($p<0.05$), shown in the panels on the right. Red and blue effect size bars represent significant enrichment in SLE and RA, respectively. Literature-derived signatures in **(b-e)** underwent co-expression analyses before being used as GSVA gene sets (see Methods).

Supplementary Figure S10. Co-expression Analysis of Synovial Cell Populations in SLE and OA Samples

a Co-expression of Single Cell Fibroblasts in SLE and OA

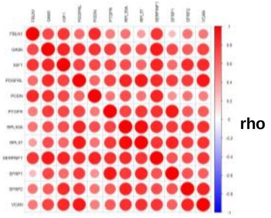

Synovial CD34⁺ sublining fibroblasts (SC-F1)

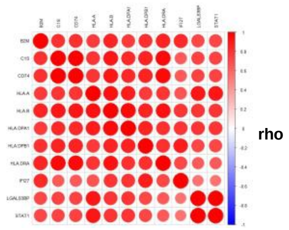

Synovial HLA-DR^{hi} sublining fibroblasts (SC-F2)

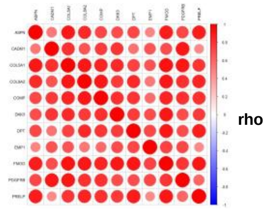

Synovial DKK3+ sublining fibroblasts (SC-F3)

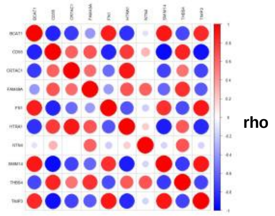

Synovial lining fibroblasts (SC-F4)

b Co-expression of Single Cell Macrophages in SLE and OA

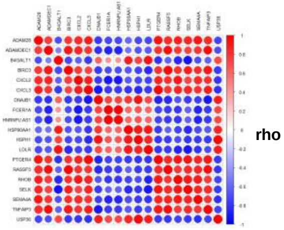

HBEGF+ proinflammatory macrophages (SC-M1)

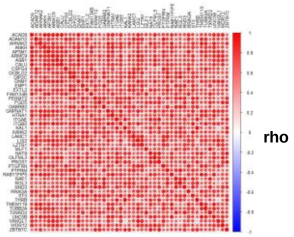

Phagocytic macrophages (SC-M2)

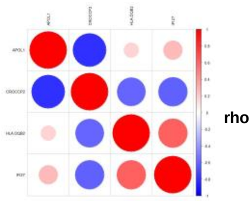

Quiescent macrophages (SC-M3)

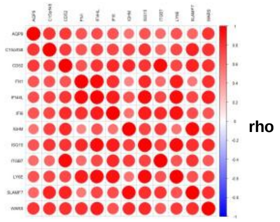

IFN-activated macrophages (SC-M4)

c Co-expression of Control Mouse Synovial Cell Populations in SLE and OA

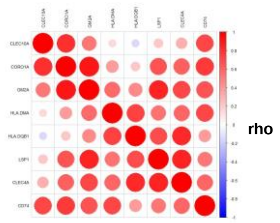

MHCII+ interstitial macrophages

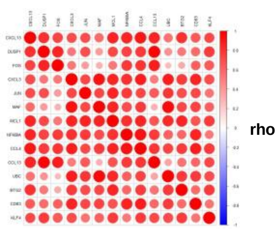

RELM-α+ interstitial macrophages

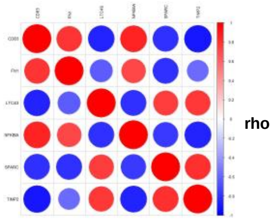

CX3CR1+ lining macrophages

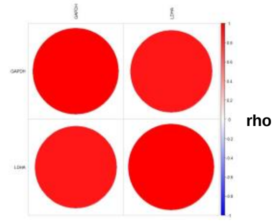

STMN1+ proliferating cells

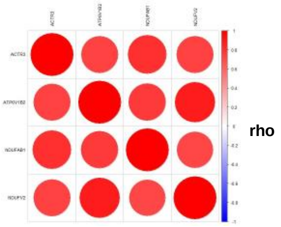

ACP5+ osteoclast precursors

d Co-expression of Mouse Synovial Cell Populations after Serum Transfer Arthritis Induction in SLE and OA

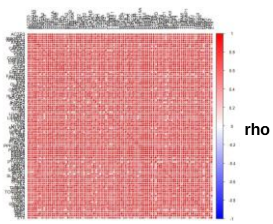

CCR2+IL1B+ infiltrating macrophages

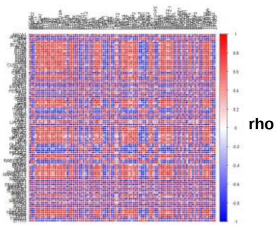

CX3CR1+ lining macrophages

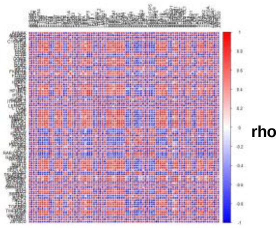

RELM-α+ interstitial macrophages

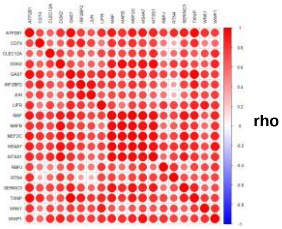

MHCII+ interstitial macrophages

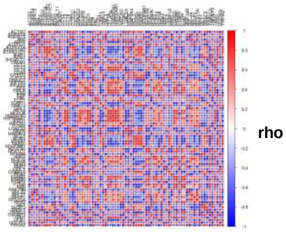

CCR2+ARG1+ infiltrating macrophages

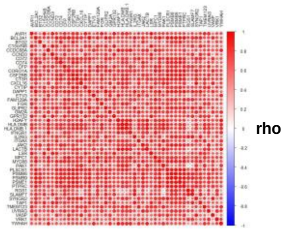

MHCII high dendritic cells

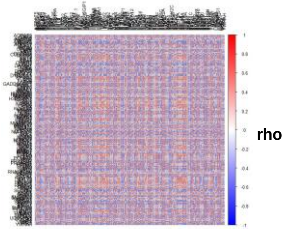

STMN1+ proliferating cells

Spearman rank correlation coefficients (rho) of gene transcripts comprising synovial tissue-specific cell signatures computed from log2 expression values in SLE and OA samples. Refined signatures shown were derived from single-cell RNA-seq analysis of synovial fibroblasts **(a)** and macrophages **(b)** [10, 11], normal mouse synovial cells **(c)** [12], and mouse synovial cells after serum transfer arthritis induction **(d)** [12]. Original signatures were culled based on percentage of significant correlations and Spearman rank rho values (see Methods).
